# Prairie plant phenology driven more by temperature than moisture in climate manipulations across a latitudinal gradient in the Pacific Northwest, USA

**DOI:** 10.1002/ece3.4995

**Published:** 2019-02-18

**Authors:** Paul B. Reed, Laurel E. Pfeifer‐Meister, Bitty A. Roy, Bart R. Johnson, Graham T. Bailes, Aaron A. Nelson, Margaret C. Boulay, Sarah T. Hamman, Scott D. Bridgham

**Affiliations:** ^1^ Institute of Ecology and Evolution University of Oregon Eugene Oregon; ^2^ Environmental Studies Program University of Oregon Eugene Oregon; ^3^ Department of Landscape Architecture University of Oregon Eugene Oregon; ^4^ Center for Natural Lands Management Olympia Washington

**Keywords:** climate manipulation, drought, latitudinal gradient, Mediterranean grassland, normalized difference vegetation index, Pacific Northwest, USA, phenology, prairie, soil moisture, warming

## Abstract

Plant phenology will likely shift with climate change, but how temperature and/or moisture regimes will control phenological responses is not well understood. This is particularly true in Mediterranean climate ecosystems where the warmest temperatures and greatest moisture availability are seasonally asynchronous. We examined plant phenological responses at both the population and community levels to four climate treatments (control, warming, drought, and warming plus additional precipitation) embedded within three prairies across a 520 km latitudinal Mediterranean climate gradient within the Pacific Northwest, USA. At the population level, we monitored flowering and abundances in spring 2017 of eight range‐restricted focal species planted both within and north of their current ranges. At the community level, we used normalized difference vegetation index (NDVI) measured from fall 2016 to summer 2018 to estimate peak live biomass, senescence, seasonal patterns, and growing season length. We found that warming exerted a stronger control than our moisture manipulations on phenology at both the population and community levels. Warming advanced flowering regardless of whether a species was within or beyond its current range. Importantly, many of our focal species had low abundances, particularly in the south, suggesting that establishment, in addition to phenological shifts, may be a strong constraint on their future viability. At the community level, warming advanced the date of peak biomass regardless of site or year. The date of senescence advanced regardless of year for the southern and central sites but only in 2018 for the northern site. Growing season length contracted due to warming at the southern and central sites (~3 weeks) but was unaffected at the northern site. Our results emphasize that future temperature changes may exert strong influence on the timing of a variety of plant phenological events, especially those events that occur when temperature is most limiting, even in seasonally water‐limited Mediterranean ecosystems.

## 1 INTRODUCTION

Plant phenology, the timing of key events in plant life cycles, is shifting with climate change (Cleland, Chuine, Menzel, Mooney, & Schwartz, 2007; Menzel et al., 2006; Parmesan & Yohe, 2003). Shifts have been observed at the individual species level (Fitter & Fitter, 2002; Whittington, Tilman, Wragg, & Powers, 2015), as well as for entire plant communities (Sherry et al., 2007; Theobald, Breckheimer, & Hille Ris Lambers, 2017), through both observational and manipulative studies. At the plant population level, the first appearance of flowers as well as the timing of peak flowering has important consequences for reproductive success and population viability. Phenological shifts in flowering may create asynchronies among interacting species (Yang & Rudolph, 2010), potentially disrupting mutualisms such as pollination or seed dispersal (Rafferty, Caradonna, & Bronstein, 2015), or result in mismatches with favorable environmental conditions, increasing the potential for detrimental events such as frost damage (Inouye, 2008). Shifts in phenology may also alter demographic vital rates and influence range distributions, which in turn can have large implications for patterns of biodiversity and species extinctions or persistence (Chuine & Beaubien, 2001; Miller‐Rushing, Høye, Inouye, & Post, 2010; Parmesan & Yohe, 2003). At the community level, changes to the timing of seasonal biomass growth and senescence can affect processes such as primary productivity, carbon cycling, and competition (Cleland et al., 2007; Tang et al., 2016).

Despite ample evidence of recent phenological shifts, the controls of future shifts are not well understood. Temperature is typically viewed as one of the strongest controls of plant phenology, although other abiotic factors such as photoperiod and moisture can also exert influences (Moore, Lauenroth, Bell, & Schlaepfer, 2015; Rathcke & Lacey, 1985). Phenological events tend to advance with warming and are generally thought to be delayed with drought (Menzel et al., 2006; Wolkovich et al., 2012), although there are conflicting reports regarding the latter (Bernal, Estiarte, & Peñuelas, 2011; Cui, Martz, & Guo, 2017). While most studies have focused on temperature, moisture may be a stronger control than temperature for late‐flowering species (Moore & Lauenroth, 2017) and is especially critical in water‐limited ecosystems (Crimmins, Crimmins, & David Bertelsen, 2010; Diez et al., 2012). In Mediterranean climate regions, which are characterized by pronounced cool/wet and warm/dry seasons, moisture becomes increasingly limiting during the latter part of the growing season. Water availability thus becomes a critical factor, and moisture manipulation has been shown to affect plant phenology within Mediterranean regions (Bernal et al., 2011; Hänel & Tielbörger, 2015). Moisture has even been shown to have greater influence on phenology than temperature in some cases, depending on the phenological event (Peñuelas et al., 2004). However, a 60‐year observational study of 29 plant species in Spain suggests temperature is the primary driver of changes in phenology in that Mediterranean region (Gordo & Sanz, 2010). The influence of biotic interactions (e.g., competition) on phenology is largely unknown outside the findings of Wolf, Zavaleta, and Selmants (2017) that plant diversity can affect phenology through its effects on soil temperature, nutrients, and moisture.

Globally, Mediterranean regions contain some of the most imperiled habitats and have among the greatest risks for biodiversity loss (Klausmeyer & Shaw, 2009; Sala et al., 2000). Much of the US Pacific Northwest (PNW) has a Mediterranean climate (Kottek, Grieser, Beck, Rudolf, & Rubel, 2006), and models for the PNW predict ~3°C temperature increases by the end of the 21st century, with increasingly warmer, wet winters and hotter, drier summers including greater drought potential during the growing season (Jung & Chang, 2012; Mote & Salathé, 2010). Native prairie ecosystems in this region have dwindled to <10% of their historic extent and most are highly degraded (Crawford & Hall, 1997; Noss, Laroe, & Scott, 1995; UFWS, 2010) because of land‐use change, altered fire regimes, and invasive species (Bachelet et al., 2011). Climate change may further exacerbate the perturbations affecting these ecosystems, causing species range shifts or contractions, declining populations, or altering biogeographic patterns (Pfeifer‐Meister et al., 2013, 2016). Considering the vulnerability of prairie species and communities within this region, it is thus imperative to explore the implications of changing temperature and moisture patterns on prairie plant phenology and abundances, so land managers can plan and adapt appropriate practices.

Several studies have demonstrated the robustness of integrating manipulative experimentation with natural climate gradients to identify climate change effects on species, communities, and ecosystems (Dunne, Harte, & Taylor, 2003; Dunne, Saleska, Fischer, & Harte, 2004; Frenne et al., 2013; Pfeifer‐Meister et al., 2013). However, this approach has been underutilized for phenological studies (but see Henry & Molau, 1997; Dunne et al., 2003; Prieto et al., 2009), especially considering that latitude may influence the magnitude or sensitivity of responses to climate change (Parmesan, 2007; Prevéy et al., 2017). Additionally, manipulative experiments designed to study climate change effects on phenology often impose extensions of the growing season via snow removal, temperature increases, or moisture manipulations (Bernal et al., 2011; Peñuelas et al., 2004; Rosa et al., 2015; Tielbörger et al., 2014; Whittington et al., 2015) but rarely are designed to manipulate both temperature and moisture, despite potentially confounding effects (Wolkovich et al., 2012).

Here, we manipulated both temperature and soil moisture in three prairies across a 520 km latitudinal Mediterranean climate gradient within the PNW to examine the responses of plant phenology at both the population and community levels. At the population level, we focused on the flowering times of eight native, range‐restricted focal species that we planted within and beyond their current ranges. Additionally, as we discovered that many species had very low survival (limiting our sample size for the flowering phenology data), we also examined how site and climate impacted their abundances. At the community scale, we focused on the seasonality of growth and senescence of canopy biomass. We asked: (a) How will the phenology of individual species, as well as prairie plant communities, respond to climate change across a latitudinal gradient? (b) Will range‐restricted species’ phenological responses and flowering abundances differ in direction and/or magnitude when planted within versus beyond their current northern range limits? And, (c) will changes to soil temperature or moisture be more predictive of phenological responses?

## 2 MATERIALS AND METHODS

### 2.1 Site descriptions

The study was conducted at three sites from southwestern Oregon to central‐western Washington in the Pacific Northwest (PNW) (Supporting Information Figure S1, Table S1). The southern site is in the Klamath‐Siskiyou ecoregion of southwestern Oregon, the central site is at the southern end of the Willamette Valley ecoregion in western Oregon, and the northern site is in the Puget Trough ecoregion of central‐western Washington (U.S. EPA [Environmental Protection Agency], 2011). There is a strong climate gradient from north to south, with the northern site experiencing the coolest mean annual temperatures and most mesic summer soil moistures, the central site experiencing intermediate temperatures and soil moisture, and the southern site experiencing the warmest mean annual temperatures and driest soils in the summer (Pfeifer‐Meister et al., 2013, 2016) (Supporting Information Table S1, Figure S2).

### 2.2 Experimental design

At each site, 20 circular plots (7.1 m^2^) were randomly assigned to one of four climate treatments with five replicates each: control (ambient temperature and precipitation), warming (canopy temperature raised by 2.5°C), warming with additional precipitation (warming + ppt; plots irrigated to fully offset a warming‐induced drying effect), and drought (annual precipitation reduced by 40%). The southern and central sites were part of a previous experiment from 2010–2012 with a different set of treatments consisting of control, warming by 2.5°C, increased precipitation intensity by 20%, and warming by 2.5°C + increased precipitation intensity by 20% (Pfeifer‐Meister et al., 2013, 2016; Reynolds, Johnson, Pfeifer‐Meister, & Bridgham, 2015). However, the precipitation intensity treatments had almost no effect on either plant or ecosystem responses since most of the additional water was applied during the wet season (Pfeifer‐Meister et al., 2013, 2016; Reynolds et al., 2015). Thus, the current experiment has the same control and warming treatments at the two southernmost sites, but the enhanced precipitation intensity plots became the drought plots, and the warming plus enhanced precipitation intensity plots became the warming + ppt plots of the current experiment. The northern site was newly established for this experiment.

Warming treatments were achieved using six 2000‐W infrared heaters per plot, as described in Pfeifer‐Meister et al. (2013). The warming + ppt plots used an automated sprinkler system (with rainwater collected on site) designed to irrigate these plots for 30 min each night that the volumetric water content was below 95% of the control plot average. The drought treatment used a common fixed rain‐out shelter design, with clear acrylic shingles (MultiCraft Plastics, Eugene, OR) covering 40% of the plot area to prevent 40% of annual rainfall from reaching the plot. The acrylic material has high light transmittance, reducing microclimatic impacts such as shading concerns or temperature buffering (Gherardi & Sala, 2013; Yahdjian & Sala, 2002). The 40% reduction in annual precipitation represents an “extreme” drought, consistent with a one‐in‐100‐year event for the three sites, determined using the Precipitation Trends and Manipulation tools from Drought‐Net (Lemoine, Sheffield, Dukes, Knapp, & Smith, 2016). Drought treatments were installed in February 2016, all warming treatments initiated by summer 2016, and irrigation initiated during summer 2016. Heaters were turned off in August and September 2017 at all three sites due to fire hazard. We used dataloggers to record continuous canopy temperature, soil temperature (at 10 cm depth), and volumetric water content (to 30 cm depth) within each plot. To compare soil moisture across sites with considerably different soil characteristics, we calculated soil matric potentials as described in Saxton and Rawls (2006). See Supporting Information Figure S2 for data on soil temperature and matric potential in plots during the study. Due to heater malfunctions in one of the central‐site warming plots for a period of the 2017 growing season, we excluded data from this plot for phenological analyses occurring during that time.

Between October 2014 and January 2015, all plots at the southern and central sites were mowed and raked while the new northern plots were treated with Glyphosate 2% (a total of three times) to remove standing biomass. By February 2015, all plots were seeded with a common mix of 29 native grass and forb species found in PNW prairies (Pfeifer‐Meister et al., 2013). Additionally, in fall of both 2015 and 2016, we seeded between 80–200 seeds per species of 14 range‐restricted species within each plot for the purposes of a separate demography experiment. These species were selected for having medium to high fidelities to upland prairies with geographic range distributions within the PNW (~41–50° latitude). Due to low establishment of six of these 14 species at all sites, only eight were used as focal species in this study (Table 1). For each species and site, we used seeds from the nearest available source population. Four species (*Collinsia grandiflora*, *Festuca roemeri*, *Microseris laciniata, *and *Plectritis congesta*) had unique sources for each site; the remaining four species (*Achyracheana mollis, Plagiobothrys nothofulvus, Ranunculus austro‐oreganus, *and *Sidalcea malviflora*) had single sources for all sites.

**Table 1:** Characteristics of the eight focal species analyzed for flowering phenology observations (Jaster, Meyers, & Sundberg, 2017)

Focal species	Abbreviation	Family	Growth habit	Duration	Approximate northern range limit
*Achyrachaena mollis *Schauer	ACHMOL	Asteraceae	Forb	Annual	~43°N
*Collinsia grandiflora *Douglas ex Lindl.	COLGRA	Plantaginaceae	Forb	Annual	~50°N
*Festuca roemeri* a	FESROE	Poaceae	Grass	Perennial	~50°N
*Microseris laciniata *(Hook.) Sch. Bip. ssp. laciniata	MICLAC	Asteraceae	Forb	Perennial	~50°N
*Plagiobothrys nothofulvus *A. Gray	PLANOT	Boraginaceae	Forb	Annual	~46°N
*Plectritis congesta *(Lindl.) DC.	PLECON	Valerianaceae	Forb	Annual	~50°N
*Ranunculus austro‐oreganus* L.D. Benson	RANAUS	Ranunculaceae	Forb	Perennial	~43°N
*Sidalcea malviflora* (DC.) A. Gray ex Benth. ssp. virgata (Howell) C.L. Hitchc.	SIDMAL	Malvaceae	Forb	Perennial	~46°N
					

a Variety *roemeri* Yu. E. Alexeev at the central and northern sites; variety *Klamathensis* B.L. Wilson at the southern site.

### 2.3 Phenology data

From April to mid‐June 2017, we collected flowering and abundance data on our eight focal species on a weekly (central and northern sites) or biweekly (southern site) basis. For each forb species, we tallied the total number of open flowers (defined by the presence of exposed stamens or stigmas) within each plot. For the lone grass species (*F. roemeri*), we tallied the total number of reproductive stalks containing spikelets (hereafter considered flowers). For all species, we recorded the total number of flowering individuals. From these observations, we identified the first flowering dates (FFD) and peak flowering dates (PFD) for each species in each plot. Additionally, we calculated temperature sensitivities (change in days per °C) for each species at each site as: (phenological event date*_i_*_,warm_ – phenological event date_ambient avg_)/Δ*T*, where Δ*T* is the difference in temperature between the warmed and ambient plots, or 2.5°C.

At the community level, we regularly measured the phenology of the canopy biomass from November 2016 to August 2018 by determining the amount of live green vegetation using a handheld Crop Circle ACS‐430 sensor (Holland Scientific Inc.), which calculates the normalized difference vegetation index (NDVI) from measurements taken above each plot canopy. NDVI is an index of “greenness” on a scale of −1 to 1, with increasing values indicating a greater quantity of live biomass (Pettorelli et al., 2005). For each plot, we calculated the date of peak biomass (maximum NDVI), date of senescence, and rate of senescence for both 2017 and 2018, and the length of the growing season from fall 2017 through summer 2018. We did not include the previous growing season length since we lacked regular NDVI measurements in fall 2016. For the date of senescence, we chose the first date following peak biomass at which the NDVI was ≤80% of the peak. We calculated the rate of senescence as the slope (ΔNDVI/days) for the three sampling points with the greatest decline in NDVI. For the southern site in 2018, we only used two sampling points because senescence was so rapid that three sampling points would not have been linear. Lastly, we calculated the length of the growing season as the difference in days between the fall 2017 green‐up (the first date following the summer 2017 minimum at which the NDVI was ≥125% of the minimum) and the end of the season (the 2018 date of senescence). This timeframe represents a full growing season in this Mediterranean climate system, as vegetation growth commences with the return of the fall rains and ceases with the return of the summer drought.

### 2.4 Statistical analyses

All analyses used R version 3.3.2 (R Core Team, 2016). Site and climate treatment effects on flowering phenology were determined by analysis of variance (ANOVA), whereas significant differences among sites and climate treatments within sites were tested using Tukey's post hoc comparisons. Because the control and drought treatments never differed for either FFD or PFD (*p* ≥ 0.19) and the warming and warming + ppt treatments only marginally differed for PFD for one species (*p* = 0.07; all other cases *p* ≥ 0.15), we collapsed the climate treatments into two temperature categories: ambient (control and drought) and warming (warming and warming + ppt) and reran analyses. Due to site × warming interactions, we tested for site effects using ambient plots only. Within sites, we tested for an effect of warming using two‐tailed *t* tests. PFD data for *C. grandiflora *at the northern site were excluded due to an overwhelmingly large sample size (>500 plants per plot) which made it logistically impossible to count flowers during its peak growing period.

To test for site and climate treatment impacts on flowering abundances, we ran generalized linear models for each species, testing for the best fit among Poisson, negative‐binomial, and zero‐inflated models by comparing Akaike information criterion (AIC). We selected the model with the lowest AIC value and tested for goodness‐of‐fit with a chi‐square test. Finally, we identified significant effects using likelihood‐ratio chi‐square tests. When a significant site x climate treatment interaction was present, we repeated this process within each site to test for climate treatment effects.

We analyzed multi‐year NDVI variables (date of peak biomass, date of senescence, and rate of senescence) with repeated measures ANOVAs with site and climate treatment as between‐subject effects and year as a within‐subject effect. Following significant year interactions, we tested these variables (and 2018 growing season length) within years against site, climate treatment, and their interaction with ANOVAs. Additionally, to test for differences in NDVI on sampling dates across the duration of measurement, we conducted repeated measures ANOVAs with sampling date as a within‐subject effect. We used logit‐transformations to improve normality and Greenhouse–Geisser corrections when sphericity was violated. Following site × date interactions, we conducted repeated measures ANOVAs within each site. Lastly, following date × climate treatment interactions, we performed one‐way ANOVAs on each date. Again, we found no differences between the control and drought treatments and between the warming and warming + ppt treatments for any of these analyses (*p* > 0.10), so we collapsed to the two ambient and warming categories and reran all NDVI analyses.

Using plot‐level environmental data, we tested for trends in phenology response variables to soil temperature and moisture variables within and across sites. We excluded FFD and PFD data for *M. laciniata *and *R. austro‐oreganus *since these species only survived at a single site. We calculated annual environmental variables using the durations 15 July 2016–15 July 2017 for 2017 phenology variables and 15 July 2017–15 July 2018 for 2018 phenology variables. For temperature variables, we used mean annual (MAT), mean winter (MWT; 1 December–28 February), and mean spring (MST; 1 March–31 May) soil temperatures. For soil moisture variables, we looked at the annual number of days below wilting point (−1,500 kPa; DBWP); the mean annual matric potential (MAMP; adjusted to account for wilting point so any value <−1,500 became −1,500); and the date of first wilting point (DFWP). Strong correlations among the three temperature variables as well as the three soil moisture variables made it inappropriate to include all variables in multiple regression. Instead, for each response variable, we created 15 total models: each combination of one temperature variable with one moisture variable (nine models), and each temperature and each moisture variable alone (six models). Then for each response variable, we used the MuMIn package (Barton, 2018) to compare and rank each model using the small‐sample‐size corrected version of Akaike information criterion (AICc). Here, we report models that would be deemed equivalent based on a δAICc <2. However, we do not report two parameter models if their AICc score was greater than a model that included only one of its parameters to maintain parsimony in interpretation. Following model ranking, we compared relative variable importance values to identify the most important explanatory variable for each response variable. These values are calculated by taking the sum of the Akaike weights (*ω*) over all models that include the explanatory variable (Burnham & Anderson, 2002). While there can be cases of over‐interpreting relative variable importance values (Galipaud, Gillingham, David, & Dechaume‐Moncharmont, 2014), it is nonetheless a reliable method if the only goal is simply to identify the single most important explanatory variable relative to all others.

## 3 RESULTS

### 3.1 Reproductive plant abundances

**Figure 1:**
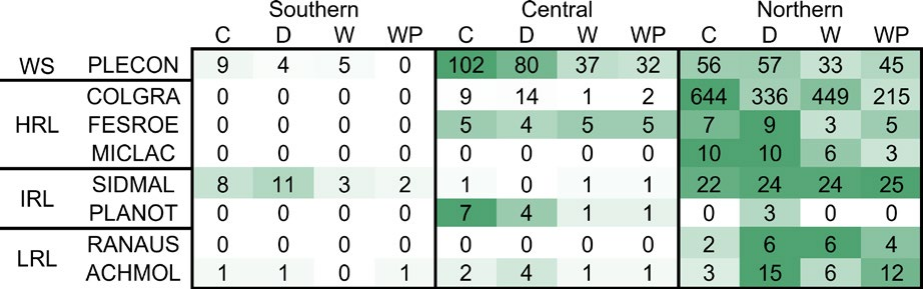
Median abundances of reproductive plants across the four climate treatments at each site. Shading is scaled independently for each species; darker corresponds to greater median abundances under that treatment and site, and lighter corresponds to lesser median abundances. C: control; D: drought; W: warming; WP: warming + ppt. Northern range‐limit groups: HRL: highest northern range limit (~50°N); IRL: intermediate northern range limit (~46°N); LRL: lowest northern range limit (~43°N; see Table 1); WS: Widespread

Site had a strong effect on focal species’ abundances. In general, the number of reproductive plants increased dramatically from south to north (Figure 1; Supporting Information Table S2). To a lesser extent, climate treatment also affected the number of reproductive plants, but effects varied considerably by species and were generally idiosyncratic within sites (Figure 1; Supporting Information Table S2). Several species had small or nonexistent reproductive populations at certain sites, within certain climate treatments, or a combination; *R. austro‐oreganus *and *M. laciniata *did not survive to reproduce at all at either the southern or central sites, nor did *F. roemeri* at the southern site. These abundance constraints ultimately hindered our ability to analyze all aspects of the flowering phenology data.

### 3.2 Flowering phenology

**Figure 2:**
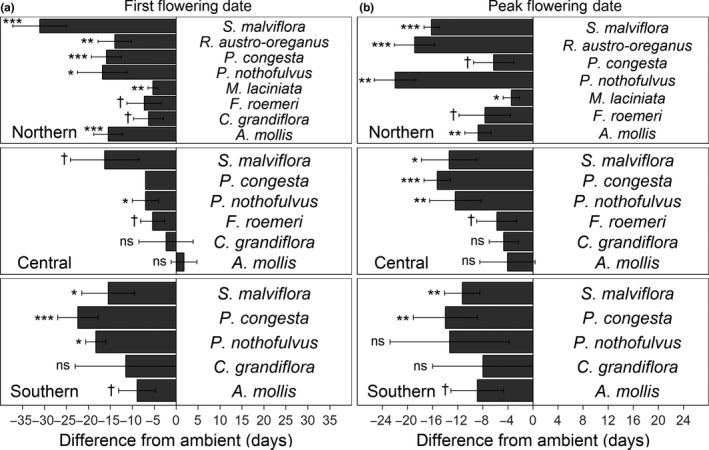
Mean difference ± standard error between warmed and ambient plots for (a) first flowering date (FFD) and (b) peak flowering date (PFD) at each site. Negative value indicates an advancement with warming. Significance codes: ns *p* > 0.1, †*p* < 0.1, *p < 0.05, **p < 0.01, ***p < 0.001; two‐tailed *t* tests. *P. congesta *FFD could not be tested statistically because it did not exhibit any variability among the warming plots (*n* = 9) nor the ambient plots (*n* = 10)

In general, warming advanced both first (FFD) and peak (PFD) flowering dates at all sites. FFD advanced under warming for four of five species at the southern site, three of five species at the central site, and all eight species at the northern site (Figure 2a; Supporting Information Table S3). A fourth species at the central site, *P. congesta, *also flowered seven days earlier in all warming plots compared to all ambient plots (Figure 2a); however, *P. congesta *did not exhibit any variability in FFD among the warming plots (*n* = 9) nor the ambient plots (*n* = 10), so we were unable to perform statistical tests on this species at this site. PFD advanced under warming relative to ambient temperature for three of five species at the southern site, four of six species at the central site*, *and all seven species with PFD data at the northern site (Figure 2b; Supporting Information Table S4).

**Figure 3:**
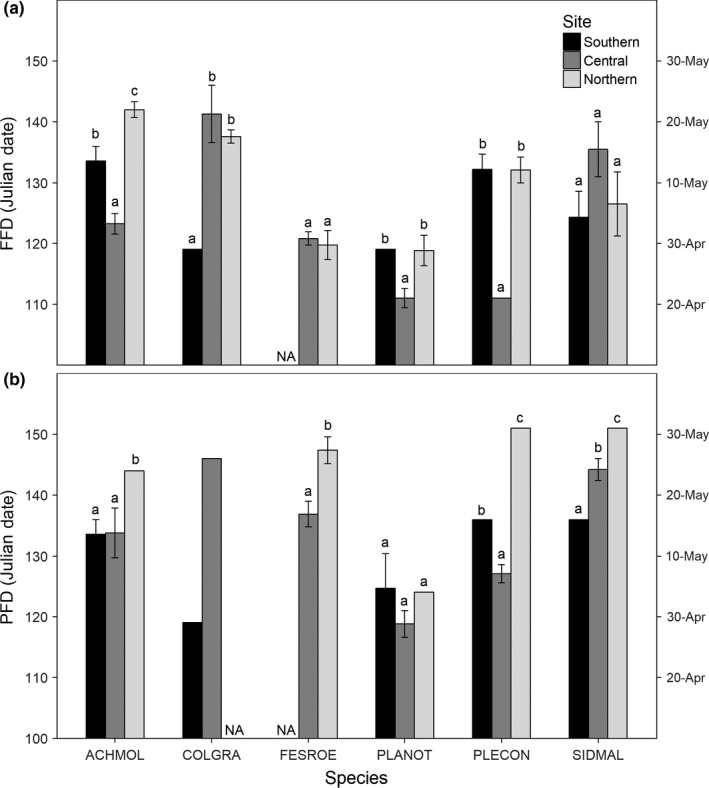
(a) First flowering date (FFD) and (b) peak flowering date (PFD) across sites, using ambient plots (due to significant site × warming interactions). Different letters indicate significant or marginal differences within a species (*p* < 0.1; Tukey's post hoc comparisons). *C. grandiflora* was not tested statistically for PFD at the northern site (see Section 2)

Under ambient temperatures, FFD and PFD varied by species across the latitudinal gradient (Figure 3). Of the annual species, *C. grandiflora *flowered earliest in the southern site, but *A. mollis, P. nothofulvus, *and *P. congesta *all flowered earliest at the central site. There was no effect of site on FFD for the two perennial species, *F. roemeri *and *S. malviflora *(Figure 3a; Supporting Information Table S3). Four species, *A. mollis*, *F. roemeri,* *P. congesta, *and *S. malviflora,* reached PFD latest in the northern site. *C. grandiflora *followed a similar trend, reaching PFD earlier at the southern site compared to the central site, but this could not be tested due to a lack of variance. One species, *P. congesta, *reached PFD earliest at the central site. Site did not significantly affect PFD for *P. nothofulvus *(Figure 3b; Supporting Information Table S4). For temperature sensitivity, *A. mollis *exhibited greater sensitivity in FFD at the northern site compared to the central (*p* = 0.003), *P. nothofulvus *at the southern site compared to the central (*p* = 0.091), *P. congesta *at both the southern and northern sites compared to the central (*p* ≤ 0.036), and *S. malviflora* at the northern site compared to both the southern and central (*p* ≤ 0.052; Supporting Information Table S5, Figure S3). PFD temperature sensitivity did not differ by site for any species (*p* > 0.10; Supporting Information Table S5, Figure S3).

We identified the most likely model(s) of temperature and moisture explanatory variables from 2016–2017 for FFD and PFD of each species (excluding *M. laciniata *and *R. austro‐oreganus*) (Supporting Information Table S6). For FFD, the most important predictors were mean winter temperature for *F. roemeri*, *P. nothofulvus*, and *S. malviflora*, mean spring temperature for *A. mollis* and *P. congesta*, and mean annual matric potential for *C. grandiflora* (Table 2; Supporting Information Figure S4). For PFD, the most important predictors were the same as for FFD for *A. mollis, P. nothofulvus, *and *S. malviflora*; for *C. grandiflora* and *F. roemeri, *the date of first wilting point, and for *P. congesta,* the mean annual temperature became the most important predictors (Table 2; Supporting Information Figure S5). For all species, temperature variables were negatively related to flowering dates; higher temperatures resulted in earlier flowering.

**Table 2:** Relative variable importance values (highest value in bold) for each phenology response variable

Phenology variable	MAT	MWT	MST	MAMP	DFWP	DBWP
FFD (2017)						
*ACHMOL*	0.007	0.010	**0.983**	0.168	0.160	0.157
*COLGRA*	0.155	0.148	0.150	**0.638**	0.228	0.129
*FESROE*	0.067	**0.690**	0.124	0.207	0.516	0.109
*PLANOT*	0.010	**0.967**	0.022	0.148	0.158	0.137
*PLECON*	0.000	0.000	**1.000**	0.272	0.655	0.069
*SIDMAL*	0.007	**0.962**	0.021	0.195	0.195	0.221
PFD (2017)						
*ACHMOL*	0.460	0.011	**0.528**	0.234	0.337	0.135
*COLGRA*	0.239	0.177	0.125	0.292	**0.705**	0.003
*FESROE*	0.556	0.065	0.362	0.091	**0.688**	0.066
*PLANOT*	0.000	**0.999**	0.001	0.150	0.460	0.113
*PLECON*	**0.948**	0.000	0.052	0.138	0.829	0.014
*SIDMAL*	0.001	**0.993**	0.007	0.540	0.408	0.048
NDVI (2017 + 2018)						
Date of peak biomass	**1.000**	0.000	0.000	0.247	0.573	0.090
Date of senescence	**0.997**	0.000	0.003	0.593	0.108	0.295
Rate of senescence	0.745	0.123	0.036	**0.823**	0.170	0.007
GSL (2018)	**0.970**	0.001	0.030	0.459	0.160	0.381

Notes

FFD: first flowering date; GSL: growing season length; PFD: peak flowering date.

Temperature variables: MAT: mean annual temp; MST: mean spring temp; MWT: mean winter temp. Moisture variables: DBWP: days below wilting point; DFWP: date of first wilting point; MAMP: mean annual matric potential.

### 3.3 Phenology of community biomass

**Figure 4:**
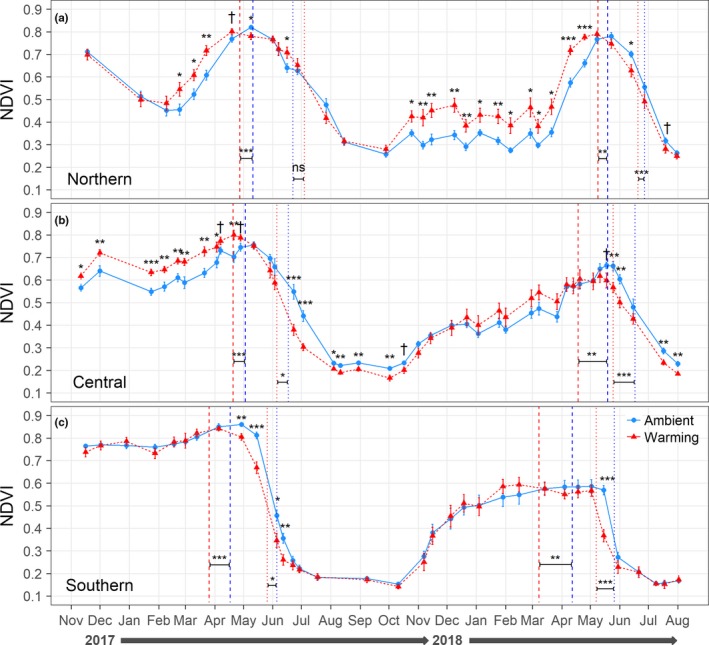
Normalized difference vegetation index (NDVI) of the ambient plots (control and drought) and warming plots (warming and warming + ppt) at each of the three sites from November 2016 to August 2018. Dates of peak biomass are shown with vertical dashed lines and dates of senescence with vertical dotted lines for both 2017 and 2018 (warming in red, ambient in blue). Significance codes: †*p* < 0.1, *p < 0.05, **p < 0.01, ***p < 0.001; two‐tailed *t* tests following repeated measures ANOVAs to examine warming effects within each date

Across all plots, peak biomass was reached 20.6 ± 4.3 days earlier under warming than under ambient temperatures, regardless of site or year (mean difference ± standard error; *p* < 0.001; Supporting Information Table S7). Peak biomass occurred earliest in the south, with the southern site reaching its peak 29.8 ± 5.3 days earlier than the central site and 38.6 ± 5.3 days earlier than the northern site (Figure 4, vertical dashed lines; *p* < 0.001).

Contrary to the results for peak biomass, the effect of warming on the date of senescence varied by site and year (site × warming and warming × year interactions: *p* < 0.05; Supporting Information Table S7). In 2017, senescence occurred 10.5 ± 3.5 and 12 ± 5.6 days earlier under warming compared to ambient at the southern and central sites, respectively (*p* ≤ 0.049). Warming did not affect the 2017 date of senescence at the northern site (*p* = 0.172; Figure 4, vertical dotted lines). In ambient plots, 2017 senescence occurred earliest in the south, with the southern site declining 12 ± 5.5 days earlier than the central site (*p* = 0.09) and 17.1 ± 5.5 days earlier than the northern site (*p* = 0.012). In 2018, senescence occurred 16.2 ± 4.3 days earlier under warming compared to ambient, regardless of site (*p* < 0.001; site x warming interaction: *p* = 0.288). The southern site senesced 20.3 ± 5.3 days earlier than the central site, which senesced 17.9 ± 5.3 days earlier than the northern site (Figure 4, vertical dotted lines; *p* ≤ 0.003).

The effect of warming on the rate (i.e., the slope) of senescence was also dependent on site and year (site × warming and site × year interactions: *p* ≤ 0.087; Supporting Information Table S7), with a greater rate of senescence under warming (−0.013 ± 0.001 Δ NDVI day^−1^) compared to ambient (−0.011 ± 0.001 Δ NDVI day^−1^) at the central site in 2017 (*p* = 0.029), but no treatment differences for either the southern or northern sites in 2017 or any site in 2018 (*p* ≥ 0.134). In ambient plots in 2017, the rate of senescence was greater in the south (−0.017 ± 0.001 Δ NDVI day^−1^) than both the central (−0.011 ± 0.001 Δ NDVI day^−1^) and northern sites (−0.010 ± 0.001 Δ NDVI day^−1^; *p* < 0.001). Similarly, in 2018 across all plots, senescence rate was again greater in the south (−0.018 ± 0.001 Δ NDVI day^−1^) than both the central (−0.011 ± 0.001 Δ NDVI day^−1^) and northern sites (−0.011 ± 0.001 Δ NDVI day^−1^; *p* < 0.001).

The effect of warming on NDVI value depended on site and date, with warming effects shifting from overall negative (suppressing biomass relative to ambient) to positive (increasing biomass relative to ambient) from south to north (Figure 4). At the southern site, warming suppressed biomass or was neutral. Suppression occurred from late April to mid‐June in 2017 and again in May 2018, during the periods of senescence in both years (Figure 4c; warming × date interaction: *p* < 0.001). At the central site, warming increased biomass from November 2016 through April 2017. By the end of June 2017, there was a shift to warming‐induced biomass suppression, which continued until mid‐October 2017, and again from mid‐May 2018 to the end of sampling in August (Figure 4b; warming × date interaction: *p* < 0.001). At the northern site, warming increased biomass from late February to mid‐April 2017, then in mid‐June, and again from late‐October 2017 to May 2018. Warming suppressed biomass relative to ambient for only one sampling date in 2017 (9 May) and only two dates in 2018 (13 June and 19 July) (Figure 4a; warming × date interaction: *p* < 0.001).

The effect of warming on the 2018 growing season length was also dependent on site (site × warming interaction: *p* = 0.076). Warming resulted in a net reduction in the southern site growing season length by 20.5 ± 6.1 days (*p* = 0.004) and in the central site growing season length by 21.6 ± 9.4 days (*p* = 0.037). There was no effect of warming on the northern site growing season length (*p* = 0.965). Under ambient conditions, the southern site had the shortest growing season (198.8 ± 4.2 days; *p* < 0.001) while the central (232.7 ± 4.2 days) and northern sites (238.4 ± 4.2 days) did not significantly differ from one another (*p* = 0.615).

For all four NDVI phenology variables, every candidate model (seven total) included mean annual temperature, while mean annual matric potential appeared in one candidate model for each variable (four models) (Supporting Information Table S6). Thus, mean annual temperature was the most important predictor for three of the four response variables (date of peak biomass, date of senescence, and 2018 growing season length), while mean annual matric potential was the most important for the rate of senescence (Table 2; Supporting Information Figure S6).

## 4 DISCUSSION

We found that changes in temperature are likely to be more impactful than changes in precipitation on many aspects of plant phenology in PNW prairies, given the expectations for potential future climatic conditions in the region. Multiple lines of evidence supported this conclusion: first, temperature variables had the highest relative importance values for 12 of 16 phenological observations (both population and community levels) modeled against an equal number of temperature and moisture predictor variables. Furthermore, none of the population‐ or community‐level variables had different responses to control (ambient temperature and precipitation) versus drought (ambient temperature and −40% precipitation), nor did warming (+2.5°C and reduced soil moisture) ever differ from warming + ppt (+2.5°C and ambient soil moisture).

Our 2.5°C increase in temperature in the warming treatments reflects expected future temperatures for the region, with models projecting ~3°C increase by the end of the 21st century (Mote & Salathé, 2010). Precipitation projections for the PNW are less certain, but generally predict an enhanced seasonality of wetter autumns and winters and drier summers, with a small (1%–2%) overall increase in annual precipitation (Mote & Salathé, 2010). Thus, our 40% reduction in annual precipitation in the drought treatment is more extreme than current predictions, yet it did not affect the phenological variables we assessed. This may be at least partly because our drought treatment had little measurable impact on soil moisture except during the beginning (i.e., fall) and end (i.e., late spring) of the growing season (Supporting Information Figure S2; see discussion below). Furthermore, the fact that the effects of our warming + ppt treatment did not differ from those of warming alone directly implicates the importance of increasing temperature. While the warming treatment was accompanied by a strong decrease in soil moisture (Supporting Information Figure S2), the warming + ppt treatment decoupled warming from the indirect effect of reducing soil moisture, indicating that the experimental effects we observed were indeed due to increasing temperature.

It is important to place our results that changes in temperature are likely to be more impactful than changes in soil moisture under future climate in the context of a Mediterranean climate system. Many prairie plants that are adapted to Mediterranean climates limit the timing of their reproductive events to the spring, prior to the extremely water‐limited summer months. Plant growth and canopy development follow a similar trend. Thus, we propose that plants adapted to a Mediterranean climate are predisposed to temperature regulation for many aspects of their phenology. Our soil moisture data provide evidence that PNW Mediterranean ecosystems are buffered against large relative changes in precipitation during much of the year. From late fall to early spring, rain was frequent enough that the soils remained near saturation point (0 kPa) regardless of climate treatment. Over the summer, however, drought severity was so extreme that the soils remained well below permanent wilting point (−1,500 kPa), also regardless of treatment. These observations held true for both 2017 and 2018, which were relatively wet and dry years, respectively, for the southern and central sites, and relatively wet and average years for the northern site. From August 2016 to August 2017, precipitation for our southern, central, and northern sites was 163%, 119%, and 132%, respectively, of the 30‐year averages from 1981–2010, while from August 2017 to August 2018, precipitation was 78%, 74%, and 106% of average, respectively (PRISM). Despite this high interannual variability in precipitation, we still saw the strong influence of temperature on community‐level phenology across years, even though annual mean temperatures during this time were no greater than ±0.5°C of the 30‐year averages for each site (PRISM). Thus, climate change would need to considerably alter the *timing* of future wet/dry seasons (i.e., substantially delaying the first rains or advancing the summer drought), rather than simply the magnitude of total precipitation, for moisture regime changes to meaningfully impact the timing of many phenological events in this system.

It is also important to note that different phenological events are likely to have different mechanistic triggers, especially in a Mediterranean climate system in which high temperatures are asynchronous with the wet season. For example, Peñuelas et al. (2004) found precipitation to be less influential than temperature on leaf‐unfolding and flowering date events yet found a stronger influence for precipitation on fruiting events in a Mediterranean shrubland in the Iberian Peninsula. Additionally, the timing of the fall green‐up in PNW prairies appears to be strongly controlled by the return of the rainy season. Indeed, we did not analyze fall 2017 green‐up with our own NDVI data because it occurred in most plots as soon as soil matric potential returned to above wilting point in mid‐October 2017, so there was not enough variation to analyze (Supporting Information Figure S2; Figure 4). Thus, it is possible that some later phenological events could be influenced by changes in moisture, although most events in this system tend to occur at times when temperature is more limiting.

At the population level in 2017, all eight of our focal species experienced some degree of advancement in first flowering dates (FFD) and peak flowering dates (PFD) with warming. Warming treatments advanced FFD from 2.1 days per °C (*M. laciniata *at the northern site) to 12.4 days per °C (*S. malviflora* at the northern site), with a total mean advancement of 5.3 days per °C across species and sites. PFD advancements ranged from 1.4 days per °C (*M. laciniata *at the northern site) to 8.8 days per °C (*P. nothofulvus *at the northern site), with a total mean advancement of 4.7 days per °C. These values fall very much in line with evidence and predictions from other studies suggesting flowering times advance on average at a rate of ~2–7.5 days per °C (Amano, Smithers, Sparks, & Sutherland, 2010; Menzel et al., 2006; Moore & Lauenroth, 2017; Wolkovich et al., 2012). Furthermore, our results are consistent with those of a recent long‐term dataset (57 years) on 115 plant species in Oregon's Willamette Valley which found that spring phenological events advanced by 5–7 days per °C (Lindh, McGahan, & Bluhm, 2018). While the flowering data we present includes only one season, we have supplemental evidence that bolsters our conclusions from independent undergraduate projects for 2016 and 2018 at our central site and for 2016 at our southern site. In 2016, the three species studied at the southern site flowered first under warming compared to the ambient temperature plots (Kanner, McCullough, & Nock, 2017), while at the central site, seven of nine species flowered first under warming (ELP, 2016 Team, unpublished data). In 2018 at the central site, low flowering‐plant abundances largely led to nonsignificant findings, although *S. malviflora* and *P. congesta* flowering times advanced under warming (ELP 2018 Team, unpublished data). Thus, the flowering phenology results presented here have largely been consistent during other years of this experiment.

Some studies have found that phenological temperature sensitivity is greater at higher latitude (Prevéy et al., 2017), whereas others have found the opposite (Wang, Ge, Dai, & Tao, 2015) or no effect (Parmesan, 2007; Wolkovich et al., 2012). We did not find consistent evidence for any type of latitudinal trend in temperature sensitivity across our sites. Of the four instances in which we found significant site effects, two cases (FFD for *A. mollis *and *S. malviflora*) exhibited greater sensitivity at the northern site, but two other cases (FFD for *P. nothofulvus* and *P. congesta*) exhibited greater sensitivity at the southern site.

We also did not find particularly strong evidence for a consistent directionality along the latitudinal gradient in the flowering times of these species under ambient temperatures. Latitude is known to impact flowering times, and we expected to see species reach flowering in ambient plots later moving from south to north, due to natural differences in the climate across this gradient. However, our environmental data indicate that the central site was slightly warmer than the southern site for much of the growing season between April and June 2017 (Supporting Information Figure S2), despite the southern site being warmer on average across the year. Thus, spring temperatures did not quite follow the latitudinal gradient, which may have contributed to these results. Furthermore, our findings across sites need to be interpreted cautiously since the southern site was not sampled with the same frequency as the central and northern sites, and we had unique seed sources across sites for *C*. *grandiflora*, *F*. *roemeri*, and *P*. *congesta*. Populations from different latitudes may differ in their phenologies based on unique evolutionary responses to growing season cues (Olsson & Agren, 2002), which may also contribute to the lack of a latitude effect on temperature sensitivity for at least the species with seeds sourced uniquely for each site.

Advances in flowering times have important implications for species’ individual fitness, interactions with other species, and the assemblages of plant communities. Shifts in flowering times may desynchronize associations with pollinators, leading to lower reproductive capacity for the host plant and cascading effects at other trophic levels (Forrest & Miller‐Rushing, 2010; Miller‐Rushing et al., 2010; Rafferty et al., 2015). In our imperiled prairies, *S. malviflora* is a known nectar source for the Fender's blue butterfly (*Icaricia icarioides fenderi *[Macy]), and both *C. grandiflora *and *P. congesta *are known host plants of the Taylor's checkerspot butterfly (*Euphydryas editha taylori*), two federally listed endangered species (Schultz, Hammond, & Wilson, 2003; Schultz et al., 2011). If phenological shifts are strong enough to cause asynchronies between these butterflies’ lifecycles and the growth and flowering of these and other key plant species, there could be dramatic implications for these butterflies’ recovery. Moreover, phenological shifts in plants of interest to prairie restoration may affect the ability of practitioners to successfully accomplish common activities such as burning (Hamman, Dunwiddie, Nuckols, & Mckinley, 2011) or targeted weed control (Dennehy et al., 2011). Conservation and restoration practitioners will likely need to develop adaptive strategies and plans that consider phenological shifts in order to continue meeting management goals (Bachelet et al., 2011).

While we show that the flowering times of native prairie species are likely to advance with warming, our finding that their abundances were higher at our northern site relative to our southern may be more critical. Even after multiple years of seeding identical quantities into our plots, only a few species were able to successfully establish populations across the entire gradient, a theme that we have observed in the past (Pfeifer‐Meister et al., 2013) and that has persisted in 2018 (unpublished data). Our southern site had very few reproductive individuals for any range‐limited focal species, apart from *S. malviflora*. This suggests that factors affecting establishment are currently hindering populations at this site, despite all species being within their current ranges at that location. Previous experiments have demonstrated that this site has high nutrient availability and levels of productivity that do not differ from our central site (Pfeifer‐Meister et al., 2013, 2016; Reynolds et al., 2015). Instead, it seems likely that the extreme summer temperatures and the early‐onset of summer drought experienced in that region (Supporting Information Figure S2) make it exceptionally difficult for these species to establish from seed. Contrary to results at the southern site, most of our species established relatively high abundances of reproductive individuals under ambient conditions at our northern site, with the exceptions of *P. nothofulvus, R. austro‐oreganus, *and *A. mollis*. Interestingly, at least *R. austro‐oreganus *and *A. mollis* were most abundant in the north under warming, warming + ppt, and drought, and this site is beyond these species’ current northern range limits. Unexpectedly, these species struggled to achieve reproduction when planted at sites within their current ranges yet had no such constraints when planted north of their current range, suggesting they may need to shift their ranges northward to persist. In general, the less extreme climatic conditions and the longer growing seasons to the north seem to be more favorable for the fitness of all eight species, irrespective of their current ranges. These findings have implications for understanding species range distributions under future climates, and in a parallel demography experiment, we are actively assessing population projections for these and six additional species across this gradient. Furthermore, these findings confirm the importance of considering climate change when attempting to select proper seed sources for rare species restoration and recovery (Havens et al., 2015) and when selecting which species to include in restoration projects (Bachelet et al., 2011).

At the community scale, we found live plant biomass (NDVI) to be affected by warming in the following ways: consistent suppression at both the southern and central sites during the late spring to summer of both relatively wet (2017) and dry (2018) years, and suppression at the northern site during the summer of an average rainfall year (2018). However, we also found positive effects of warming at the central site during the winter and spring of the wet year (2017) and at the northern site during the winter, spring, and fall of both wet and average years. Thus, for the central and northern sites, there appear to be interactions between the effect of warming and annual rainfall on live biomass across parts of the year, a phenomenon that has been previously documented (Mueller et al., 2016; Zelikova et al., 2015). At the southern site, however, this interaction is absent; warming is consistently negative. The impacts of future climate change on aboveground prairie biomass thus appear to depend substantially on the position of each site across a latitudinal gradient of increasingly severe Mediterranean drought.

Furthermore, we saw a warming‐induced reduction of the 2018 growing season length at both the southern and central sites but a neutral effect at the northern site. Growing season lengths at higher northern latitudes (>45°N) are reported to be increasing with global warming (Ibáñez et al., 2010; Tucker et al., 2001), yet the results from our northern site (~47°N) show no effect, at least in 2018. Gordo and Sanz (2009) reported increases in growing season lengths with warming in another Mediterranean climate system (Spain), which they attributed to large advancements in spring leaf‐unfolding dates and smaller advancements in autumn leaf‐falling dates. The contractions in growing season lengths at our southern and central sites likely reflect the fact that warming considerably advanced the date of senescence at these two sites, whereas it only caused a small advancement at the northern site (Figure 4). Cui et al. (2017) also found contractions in growing season length in their Canadian prairie systems but attributed their findings to moisture limitations and not warming. Because we lacked NDVI data from fall 2016, we were unable to encapsulate the entirety of the 2017 growing season. However, considering the 2017 date of senescence advanced with warming at the southern and central sites, it is likely that warming would have also reduced the length of the 2017 growing season at these two sites. Contrarily, there may have been either a neutral or slight positive effect of warming on the 2017 growing season length at the northern site, considering its senescence date was unaffected by warming.

Shifts in the phenology of canopy biomass may have implications for community dynamics and ecosystem processes. Changes to growing season lengths are known to affect water cycling, rates and amount of carbon sequestration, and nutrient uptake from the soil (Ibáñez et al., 2010). Shorter growing seasons could reduce annual productivity, thus lessening current rates of CO_2_ sequestration (Cleland et al., 2007). Additionally, shifting community biomass phenology may provide chances for exotic species to seize on resource opportunities previously unavailable to them, increasing the potential for community invasions (Prevéy & Seastedt, 2014). Moreover, these phenological shifts could lead to greater fire hazard during the dry season. In our experiment, we saw cases of biomass increasing with warming in the winter, meaning there could be a greater accumulation of herbaceous fuels. When this is followed by an earlier date of senescence, warming may be expected to cause both an earlier and more extreme fire season in the US west (Westerling, Hidalgo, Cayan, & Swetnam, 2006).

Overall, our study offers substantial evidence that future changes in temperature may have great influence on the timing of many key plant phenological events in a Mediterranean climate system and that effects due to changes in soil moisture may be buffered from even large changes in the amount of precipitation so long as the timing and duration of the rainy season are unchanged. We observed a strong influence of temperature on flowering phenology in eight native plant species both within and beyond their current geographic ranges, as well as for canopy biomass phenology at the community scale. Additionally, we found that the majority of our eight focal species are experiencing considerable reductions in their abundances near or south of their northern range limits, suggesting that the clock is ticking on their ability to persist within their current ranges.

## CONFLICT OF INTEREST

None declared.

## AUTHORS’ CONTRIBUTIONS

PR analyzed the data and wrote the manuscript. SB, LPM, BR, BJ, and PR designed the study. All authors contributed to data collection and revisions, with substantial edits made by SB, LPM, BR, BJ, and SH.

## Data Availability

Data are available at the Dryad Digital Repository: https://doi.org/10.5061/dryad.rg0n5t5.

## References

[ece34995-bib-0001] Amano, T. , Smithers, R. J. , Sparks, T. H. , & Sutherland, W. J. (2010). A 250‐year index of first flowering dates and its response to temperature changes. Proceedings of the Royal Society B: Biological Sciences, 277(1693), 2451–2457. 10.1098/rspb.2010.0291 PMC2894925 20375052

[ece34995-bib-0002] Bachelet, D. , Johnson, B. R. , Bridgham, S. D. , Dunn, P. V. , Anderson, H. E. , & Rogers, B. M. (2011). Climate change impacts on western Pacific Northwest prairies and savannas. Northwest Science, 85(2), 411–429. 10.3955/046.085.0224

[ece34995-bib-0003] Barton, K. (2018). MuMIn: Multi‐Model Inference. R package version 1.40.4. Retrived from https://CRAN.R-project.org/package=MuMIn

[ece34995-bib-0004] Bernal, M. , Estiarte, M. , & Peñuelas, J. (2011). Drought advances spring growth phenology of the Mediterranean shrub Erica multiflora. Plant Biology, 13(2), 252–257. 10.1111/j.1438-8677.2010.00358.x 21309971

[ece34995-bib-0005] Burnham, K. P. , & Anderson, D. R . (2002). Model selection and multimodel inference: a practical information‐theoretic approach (second edition). Ecological Modelling, 172, 96–97.

[ece34995-bib-0006] Chuine, I. , & Beaubien, E. G. (2001). Phenology is a major determinant of tree species range. Ecology Letters, 4, 500–510. 10.1046/j.1461-0248.2001.00261.x

[ece34995-bib-0007] Cleland, E. E. , Chuine, I. , Menzel, A. , Mooney, H. A. , & Schwartz, M. D. (2007). Shifting plant phenology in response to global change. Trends in Ecology and Evolution, 22(7), 357–365. 10.1016/j.tree.2007.04.003 17478009

[ece34995-bib-0008] Crawford, R. , & Hall, H. (1997). Changes in the south Puget prairie landscape In Dunn P., & Ewing K. (Eds.), Ecology and conservation of the South Puget Sound Prairie Landscape (pp. 11–15). Seattle, WA: The Nature Conservancy.

[ece34995-bib-0009] Crimmins, T. M. , Crimmins, M. A. , & David Bertelsen, C. (2010). Complex responses to climate drivers in onset of spring flowering across a semi‐arid elevation gradient. Journal of Ecology, 98(5), 1042–1051. 10.1111/j.1365-2745.2010.01696.x

[ece34995-bib-0010] Cui, T. , Martz, L. , & Guo, X. (2017). Grassland phenology response to drought in the Canadian prairies. Remote Sensing, 9(1258), 1–21.

[ece34995-bib-0011] Dennehy, C. , Alverson, E. R. , Anderson, H. E. , Clements, D. R. , Gilbert, R. , & Kaye, T. N. (2011). Management strategies for invasive plants in Pacific Northwest Prairies, Savannas, and Oak Woodlands. Northwest Science, 85(2). 10.3955/046.085.0219

[ece34995-bib-0012] Diez, J. M. , Ibáñez, I. , Miller‐Rushing, A. J. , Mazer, S. J. , Crimmins, T. M. , Crimmins, M. A. , … Inouye, D. W. (2012). Forecasting phenology: From species variability to community patterns. Ecology Letters, 15(6), 545–553. 10.1111/j.1461-0248.2012.01765.x 22433120

[ece34995-bib-0013] Dunne, J. A. , Harte, J. , & Taylor, K. J. (2003). Subalpine meadow flowering phenology responses to climate change: Integrating experimental and gradient methods. Ecological Monographs, 73(1), 69–86. 10.1890/0012-9615(2003)073[0069:SMFPRT]2.0.CO;2

[ece34995-bib-0014] Dunne, J. A. , Saleska, S. R. , Fischer, M. L. , & Harte, J. (2004). Integrating experimental and gradient methods in ecological climate change research. Ecology, 85(4), 904–916. 10.1890/03-8003

[ece34995-bib-0015] ELP (Environmental Leadership Program) 2016 and 2018 Teams. (2016). University of Oregon. Unpublished data. Retrieved from https://blogs.uoregon.edu/phenology/results/

[ece34995-bib-0016] Fitter, A. H. , & Fitter, R. S. R. (2002). Rapid changes in flowering time in British plants. Science, 296, 1689–1691. 10.1126/science.1071617 12040195

[ece34995-bib-0017] Forrest, J. , & Miller‐Rushing, A. J. (2010). Toward a synthetic understanding of the role of phenology in ecology and evolution. Philosophical Transactions of the Royal Society B: Biological Sciences, 365, 3101–3112. 10.1098/rstb.2010.0145 PMC2981948 20819806

[ece34995-bib-0018] Frenne, P. D. , Graae, B. J. , Rodríguez‐Sanchez, F. , Kolb, A. , Chabrerie, O. , Decocq, G. , … Verheyen, K. (2013). Latitudinal gradients as natural laboratories to infer species’ responses to temperature. Journal of Ecology, 101, 784–795. 10.1111/1365-2745.12074

[ece34995-bib-0019] Galipaud, M. , Gillingham, M. A. F. , David, M. , & Dechaume‐Moncharmont, F. X. (2014). Ecologists overestimate the importance of predictor variables in model averaging: A plea for cautious interpretations. Methods in Ecology and Evolution, 5, 983–991. 10.1111/2041-210X.12251

[ece34995-bib-0020] Gherardi, L. A. , & Sala, O. E. (2013). Automated rainfall manipulation system: A reliable and inexpensive tool for ecologists. Ecosphere, 4(2), 1–10. 10.1890/ES12-00371.1

[ece34995-bib-0021] Gordo, O. , & Sanz, J. J. (2009). Long‐term temporal changes of plant phenology in the Western Mediterranean. Global Change Biology, 15, 1930–1948. 10.1111/j.1365-2486.2009.01851.x

[ece34995-bib-0022] Gordo, O. , & Sanz, J. J. (2010). Impact of climate change on plant phenology in Mediterranean ecosystems. Global Change Biology, 16, 1082–1106. 10.1111/j.1365-2486.2009.02084.x

[ece34995-bib-0023] Hamman, S. T. , Dunwiddie, P. W. , Nuckols, J. L. , & Mckinley, M. (2011). Fire as a restoration tool in Pacific Northwest prairies and oak woodlands: Challenges, successes, and future directions. Northwest Science, 85(2), 317–328. 10.3955/046.085.0218

[ece34995-bib-0024] Hänel, S. , & Tielbörger, K. (2015). Phenotypic response of plants to simulated climate change in a long‐term rain‐manipulation experiment: A multi‐species study. Oecologia, 177, 1015–1024. 10.1007/s00442-015-3231-8 25707776

[ece34995-bib-0025] Havens, K. , Vitt, P. , Still, S. , Kramer, A. T. , Fant, J. B. , & Schatz, K. (2015). Seed sourcing for restoration in an era of climate change. Natural Areas Journal, 35(1), 122–133. 10.3375/043.035.0116

[ece34995-bib-0026] Henry, G. H. R. , & Molau, U. (1997). Tundra plants and climate change: The International Tundra Experiment (ITEX). Global Change Biology, 3(S1), 1–9. 10.1111/j.1365-2486.1997.gcb132.x

[ece34995-bib-0027] Ibáñez, I. , Primack, R. B. , Miller‐Rushing, A. J. , Ellwood, E. , Higuchi, H. , Lee, S. D. , … Silander, J. A. (2010). Forecasting phenology under global warming. Philosophical Transactions of the Royal Society B: Biological Sciences, 365, 3247–3260. 10.1098/rstb.2010.0120 PMC2981942 20819816

[ece34995-bib-0028] Inouye, D. W. (2008). Effects of climate change on phenology, frost damage, and floral abundance of montane wildflowers. Ecology, 89(2), 353–362. 10.1890/06-2128.1 18409425

[ece34995-bib-0029] Jaster, T. , Meyers, S. , & Sundberg, S. (Eds.) (2017). Oregon vascular plant checklist. [Family]. Retrieved from http://www.oregonflora.org/checklist.php. Version 1.7

[ece34995-bib-0030] Jung, I. W. , & Chang, H. (2012). Climate change impacts on spatial patterns in drought risk in the Willamette River Basin, Oregon, USA. Theoretical and Applied Climatology, 108, 355–371.

[ece34995-bib-0031] Kanner, M. , McCullough, L. , & Nock, K. (2017). Springing forward: Changes in phenology of native plant species in Southern Oregon prairies as a result of experimental climate change. Oregon Undergraduate Research Journal, 11(1). 10.5399/uo/ourj.11.1.5

[ece34995-bib-0032] Klausmeyer, K. R. , & Shaw, M. R. (2009). Climate change, habitat loss, protected areas and the climate adaptation potential of species in mediterranean ecosystems worldwide. PLoS ONE, 4(7), e6392 10.1371/journal.pone.0006392 19641600 PMC2712077

[ece34995-bib-0033] Kottek, M. , Grieser, J. , Beck, C. , Rudolf, B. , & Rubel, F. (2006). World map of the Köppen‐Geiger climate classification updated. Meteorologische Zeitschrift, 15(3), 259–263. 10.1127/0941-2948/2006/0130

[ece34995-bib-0034] Lemoine, N. P. , Sheffield, J. , Dukes, J. S. , Knapp, A. K. , & Smith, M. D. (2016). Terrestrial Precipitation Analysis (TPA): A resource for characterizing long‐term precipitation regimes and extremes. Methods in Ecology and Evolution, 7(11), 1396–1401. 10.1111/2041-210X.12582

[ece34995-bib-0035] Lindh, B. C. , McGahan, K. A. , & Bluhm, W. L. (2018). Changes in urban plant phenology in the Pacific Northwest from 1959 to 2016: Anthropogenic warming and natural oscillation. International Journal of Biometeorology, 62(9), 1675–1684. 10.1007/s00484-018-1567-6 29911283

[ece34995-bib-0036] Menzel, A. , Sparks, T. H. , Estrella, N. , Koch, E. , Aaasa, A. , Ahas, R. , … Zust, A. (2006). European phenological response to climate change matches the warming pattern. Global Change Biology, 12, 1969–1976. 10.1111/j.1365-2486.2006.01193.x

[ece34995-bib-0037] Miller‐Rushing, A. J. , Høye, T. T. , Inouye, D. W. , & Post, E. (2010). The effects of phenological mismatches on demography. Philosophical Transactions of the Royal Society B: Biological Sciences, 365, 3177–3186. 10.1098/rstb.2010.0148 PMC2981949 20819811

[ece34995-bib-0038] Moore, L. M. , & Lauenroth, W. K. (2017). Differential effects of temperature and precipitation on early‐ vs. late‐flowering species. Ecosphere, 8(5), 1–18.29552374

[ece34995-bib-0039] Moore, L. M. , Lauenroth, W. K. , Bell, D. M. , & Schlaepfer, D. R. (2015). Soil water and temperature explain canopy phenology and onset of spring in a semiarid steppe. Great Plains Research, 25(2), 121–138. 10.1353/gpr.2015.0027

[ece34995-bib-0040] Mote, P. W. , & Salathé, E. P. (2010). Future climate in the Pacific Northwest. Climatic Change, 102(1), 29–50. 10.1007/s10584-010-9848-z

[ece34995-bib-0041] Mueller, K. E. , Blumenthal, D. M. , Pendall, E. , Carrillo, Y. , Dijkstra, F. A. , Williams, D. G. , … Penuelas, J. (2016). Impacts of warming and elevated CO2 on a semi‐arid grassland are non‐additive, shift with precipitation, and reverse over time. Ecology Letters, 19(8), 956–966. 27339693 10.1111/ele.12634

[ece34995-bib-0042] Noss, R. F. , Laroe, E. T. III , & Scott, J. M. (1995). Endangered ecosystems of the United States: A preliminary assessment of loss and degradation. Washington, DC: National Biological Service.

[ece34995-bib-0043] Olsson, K. , & Agren, J. (2002). Latitudinal population differentiation in phenology, life history and flower morphology in the perennial herb Lythrum salicaria. Journal of Evolutionary Biology, 15, 983–996.

[ece34995-bib-0044] Parmesan, C. (2007). Influences of species, latitudes and methodologies on estimates of phenological response to global warming. Global Change Biology, 13, 1860–1872. 10.1111/j.1365-2486.2007.01404.x

[ece34995-bib-0045] Parmesan, C. , & Yohe, G. (2003). A globally coherent fingerprint of climate change impacts across natural systems. Nature, 421, 37–42. 10.1038/nature01286 12511946

[ece34995-bib-0046] Peñuelas, J. , Filella, I. , Zhang, X. , Llorens, L. , Ogaya, R. , Lloret, F. , … Terradas, J. (2004). Complex spatiotemporal phenological shifts as a response to rainfall changes. New Phytologist, 161(3), 837–846. 10.1111/j.1469-8137.2004.01003.x 33873715

[ece34995-bib-0047] Pettorelli, N. , Vik, J. O. , Mysterud, A. , Gaillard, J. , Tucker, C. J. , Stenseth, N. C. , & Lyon, C. B. (2005). Using the satellite‐derived NDVI to assess ecological responses to environmental change. Trends in Ecology and Evolution, 20(9), 503–510. 10.1016/j.tree.2005.05.011 16701427

[ece34995-bib-0048] Pfeifer‐Meister, L. , Bridgham, S. D. , Little, C. J. , Reynolds, L. L. , Goklany, M. E. , & Johnson, B. R. (2013). Pushing the limit: Experimental evidence of climate effects on plant range distributions. Ecology, 94(10), 2131–2137. 10.1890/13-0284.1 24358697

[ece34995-bib-0049] Pfeifer‐Meister, L. , Bridgham, S. D. , Reynolds, L. L. , Goklany, M. E. , Wilson, H. E. , Little, C. J. , … Johnson, B. R. (2016). Climate change alters plant biogeography in Mediterranean prairies along the West Coast, USA. Global Change Biology, 22, 845–855. 10.1111/gcb.13052 26222331

[ece34995-bib-0050] Prevéy, J. S. , & Seastedt, T. R. (2014). Seasonality of precipitation interacts with exotic species to alter composition and phenology of a semi‐arid grassland. Journal of Ecology, 102, 1549–1561. 10.1111/1365-2745.12320

[ece34995-bib-0051] Prevéy, J. , Vellend, M. , Ruger, N. , Hollister, R. D. , Bjorkman, A. D. , Myers‐Smith, I. H. , … Rixen, C. (2017). Greater temperature sensitivity of plant phenology at colder sites: Implications for convergence across northern latitudes. Global Change Biology, 23, 2660–2671. 10.1111/gcb.13619 28079308

[ece34995-bib-0052] Prieto, P. , Peñuelas, J. , Niinemets, Ü. , Ogaya, R. , Schmidt, I. K. , Beier, C. , … Estiarte, M. (2009). Changes in the onset of spring growth in shrubland species in response to experimental warming along a north‐south gradient in Europe. Global Ecology and Biogeography, 18(4), 473–484. 10.1111/j.1466-8238.2009.00460.x

[ece34995-bib-0053] PRISM Climate Group . Oregon State University. Retrieved from http://prism.oregonstate.edu, created 4 Feb 2004.

[ece34995-bib-0054] R Core Team (2016). R: A language and environment for statistical computing. Vienna, Austria: R Foundation for Statistical Computing Retrieved from https://www.R-project.org/

[ece34995-bib-0055] Rafferty, N. E. , Caradonna, P. J. , & Bronstein, J. L. (2015). Phenological shifts and the fate of mutualisms. Oikos, 124(1), 14–21. 10.1111/oik.01523 25883391 PMC4396844

[ece34995-bib-0056] Rathcke, B. , & Lacey, E. P. (1985). Phenological patterns of terrestrial plants. Annual Review of Ecology, Evolution, and Systematics, 16, 179–214. 10.1146/annurev.es.16.110185.001143

[ece34995-bib-0057] Reynolds, L. L. , Johnson, B. R. , Pfeifer‐Meister, L. , & Bridgham, S. D. (2015). Soil respiration response to climate change in Pacific Northwest prairies is mediated by a regional Mediterranean climate gradient. Global Change Biology, 21, 487–500. 10.1111/gcb.12732 25205511

[ece34995-bib-0058] Rosa, R. K. , Oberbauer, S. F. , Starr, G. , La Puma, I. P. , Pop, E. , Ahlquist, L. , & Baldwin, T. (2015). Plant phenological responses to a long‐term experimental extension of growing season and soil warming in the tussock tundra of Alaska. Global Change Biology, 21, 4520–4532. 10.1111/gcb.13040 26183112

[ece34995-bib-0059] Sala, O. E. , Chapin, F. S. III , Armesto, J. J. , Berlow, E. , Bloomfield, J. , Dirzo, R. , … Wall, D. H. (2000). Global biodiversity scenarios for the year 2100. Science, 287, 1770–1774. 10710299 10.1126/science.287.5459.1770

[ece34995-bib-0060] Saxton, K. E. , & Rawls, W. J. (2006). Soil water characteristic estimates by texture and organic matter for hydrologic solutions. Soil Science Society of America Journal, 1578, 1569–1578. 10.2136/sssaj2005.0117

[ece34995-bib-0061] Schultz, C. B. , Hammond, P. C. , & Wilson, M. V. (2003). Biology of the Fender's blue butterfly (Icaricia icarioides fenderi Macy), an endangered species of western Oregon native prairies. Natural Areas Journal, 23(1), 61–71.

[ece34995-bib-0062] Schultz, C. B. , Henry, E. , Carleton, A. , Hicks, T. , Thomas, R. , Potter, A. , … Reader, B. (2011). Conservation of prairie‐oak butterflies in Oregon, Washington, and British Columbia. Northwest Science, 85(2), 361–388. 10.3955/046.085.0221

[ece34995-bib-0063] Sherry, R. A. , Zhou, X. , Gu, S. , Arnone, J. A. , Schimel, D. S. , Verburg, P. S. , … Luo, Y. (2007). Divergence of reproductive phenology under climate warming. Proceedings of the National Academy of Sciences of the United States of America, 104(1), 198–202. 10.1073/pnas.0605642104 17182748 PMC1713188

[ece34995-bib-0064] Tang, J. , Körner, C. , Muraoka, H. , Piao, S. , Shen, M. , Thackeray, S. J. , & Yang, X. (2016). Emerging opportunities and challenges in phenology: A review. Ecosphere, 7(8), 1–17. 10.1002/ecs2.1436

[ece34995-bib-0065] Theobald, E. J. , Breckheimer, I. , Hille Ris Lambers, J. (2017). Climate drives phenological reassembly of a mountain wild flower meadow community. Ecology, 98(11), 2799–2812. 10.1002/ecy.1996 29023677

[ece34995-bib-0066] Tielbörger, K. , Bilton, M. C. , Metz, J. , Kigel, J. , Holzapfel, C. , Lebrija‐Trejos, E. , … Sternberg, M. (2014). Middle‐Eastern plant communities tolerate 9 years of drought in a multi‐site climate manipulation experiment. Nature Communications, 5(5102), 1–9. 10.1038/ncomms6102 PMC4205856 25283495

[ece34995-bib-0067] Tucker, C. J. , Slayback, D. A. , Pinzon, J. E. , Los, S. O. , Myneni, R. B. , & Taylor, M. G. (2001). Higher northern latitude normalized difference vegetation index and growing season trends from 1982 to 1999. International Journal of Biometeorology, 45(4), 184–190. 10.1007/s00484-001-0109-8 11769318

[ece34995-bib-0068] U.S. EPA [Environmental Protection Agency] . (2011). Level III ecoregions of the conterminous United States. U.S. Corvalis, OR: EPA Office of Research and Development, National Health and Environmental Effects Research Laboratory.

[ece34995-bib-0069] U.S. Fish and Wildlife Service (2010). Recovery plan for the prairie species of western Oregon and southwestern Washington.

[ece34995-bib-0070] Wang, H. , Ge, Q. , Dai, J. , & Tao, Z. (2015). Geographical pattern in first bloom variability and its relation to temperature sensitivity in the USA and China. International Journal of Biometeorology, 59(8), 961–969. 10.1007/s00484-014-0909-2 25312515

[ece34995-bib-0071] Westerling, A. L. , Hidalgo, H. G. , Cayan, D. R. , & Swetnam, T. W. (2006). Warming and earlier spring increase Western U.S. forest wildfire activity. Science, 313(5789), 940–943. 16825536 10.1126/science.1128834

[ece34995-bib-0072] Whittington, H. R. , Tilman, D. , Wragg, P. D. , & Powers, J. S. (2015). Phenological responses of prairie plants vary among species and year in a three‐year experimental warming study. Ecosphere, 6(10), 1–15. 10.1890/ES15-00070.1

[ece34995-bib-0073] Wolf, A. A. , Zavaleta, E. S. , & Selmants, P. C. (2017). Flowering phenology shifts in response to biodiversity loss. Proceedings of the National Academy of Sciences of the United States of America, 114(13), 3463–3468. 10.1073/pnas.1608357114 28289231 PMC5380019

[ece34995-bib-0074] Wolkovich, E. M. , Cook, B. I. , Allen, J. M. , Crimmins, T. M. , Betancourt, J. L. , Travers, S. E. , … Cleland, E. E. (2012). Warming experiments underpredict plant phenological responses to climate change. Nature, 485, 8–12. 10.1038/nature11014 22622576

[ece34995-bib-0075] Yahdjian, L. , & Sala, O. E. (2002). A rainout shelter design for intercepting different amounts of rainfall. Oecologia, 133, 95–101. 10.1007/s00442-002-1024-3 28547315

[ece34995-bib-0076] Yang, L. H. , & Rudolph, V. H. W. (2010). Phenology, ontogeny and the effects of climate change on the timing of species interactions. Ecology Letters, 13, 1–10. 10.1111/j.1461-0248.2009.01402.x 19930396

[ece34995-bib-0077] Zelikova, T. J. , Williams, D. G. , Hoenigman, R. , Blumenthal, D. M. , Morgan, J. A. , & Pendall, E. (2015). Seasonality of soil moisture mediates responses of ecosystem phenology to elevated CO_2_ and warming in a semi‐arid grassland. Journal of Ecology, 103(5), 1119–1130.

