# Supplementary material for: Prairie plant phenology driven more by temperature than moisture in climate manipulations across a latitudinal gradient in the Pacific Northwest, USA
Source: Ecol Evol. 2019 Feb 18;9(6):3637–50. doi: 10.1002/ece3.4995 (PMC6434541; doi:10.1002/ece3.4995)
Supplement: Supplementary file 1 [file ECE3-9-3637-s001.pdf]

Supporting Information:

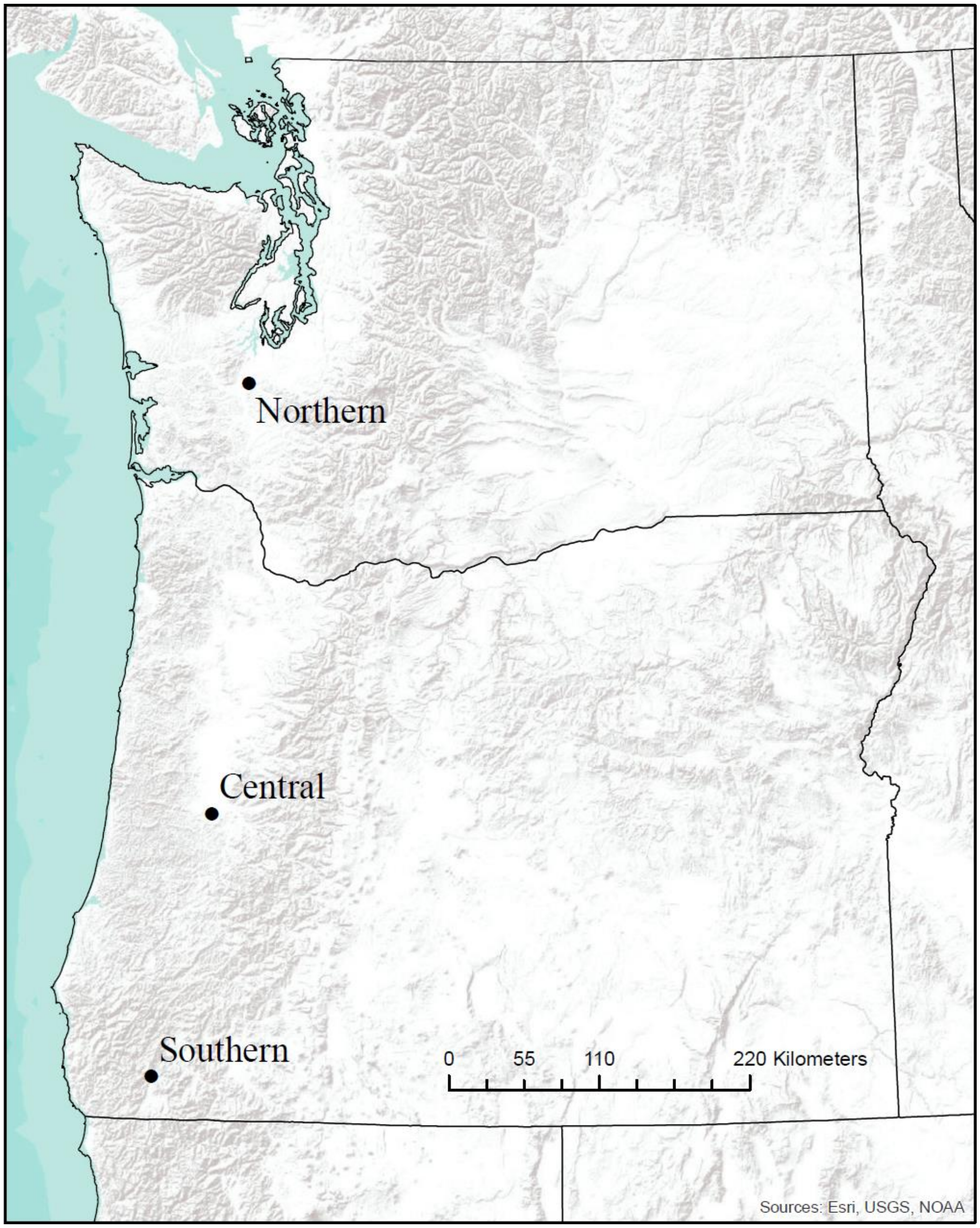

Figure S1. Locations of the three sites from southwestern Oregon to central-western Washington along the interior valleys of the Pacific Northwest. The sites span a 520 km latitudinal Mediterranean climate gradient of increasingly warmer and drier growing seasons moving from north to south. Map data sourced from Esri, USGS, NOAA, and US Census Bureau.

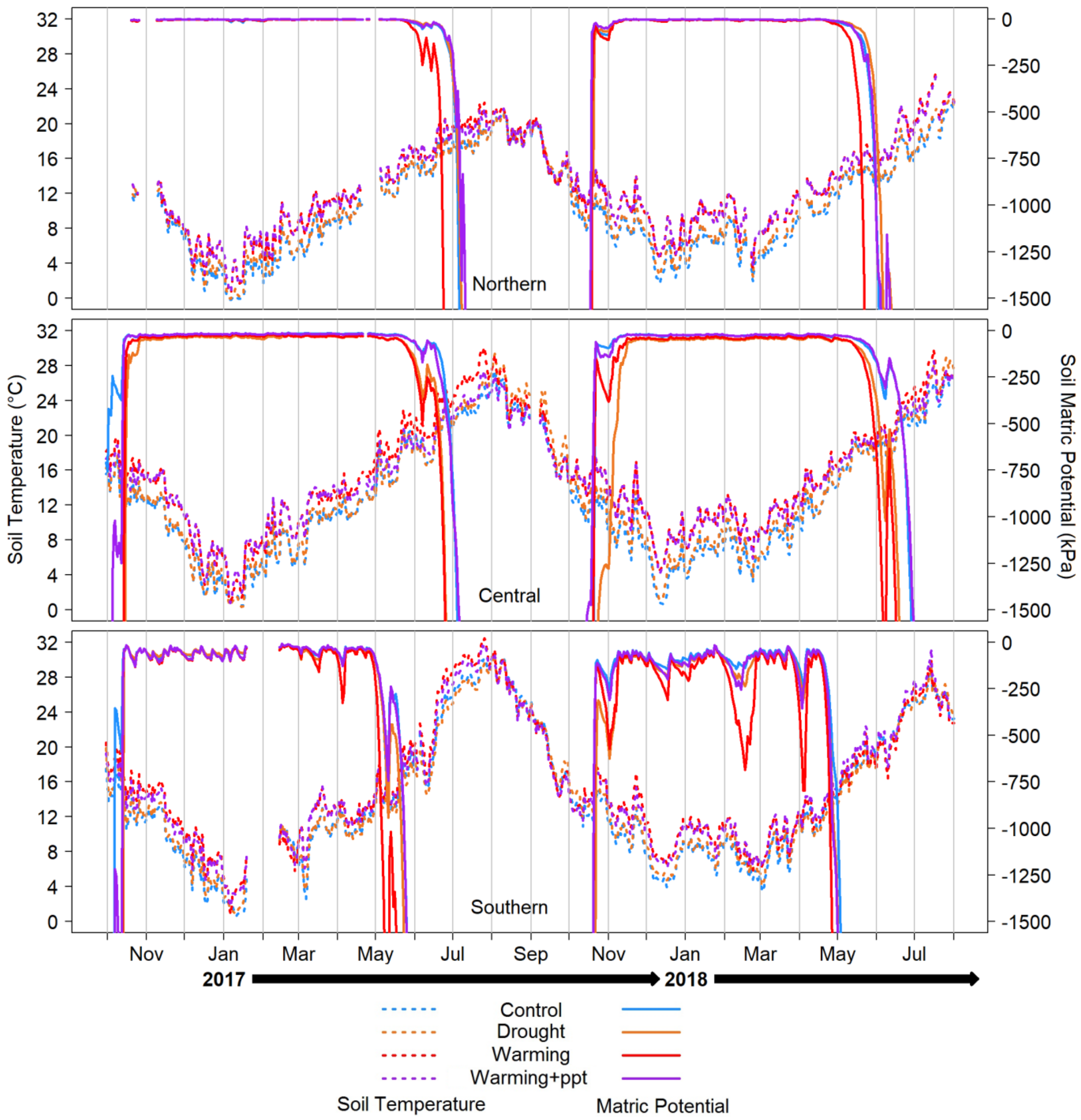

Figure S2. Mean daily soil temperature at 10 cm depth (left y-axis, dotted lines) and soil matric potential to 30 cm depth (right y-axis, solid lines) from Sept. 30, 2016 – Aug. 1, 2018 in the four climate treatments at each site. Note the earlier onset of summer drought (matric potential < -1500 kPa) and higher annual temperatures moving from north to south, and earlier summer drought in 2018 compared to 2017. Breaks in the data are due to equipment errors. Heaters were

turned off from August – October 2017 at all three sites due to fire hazards.

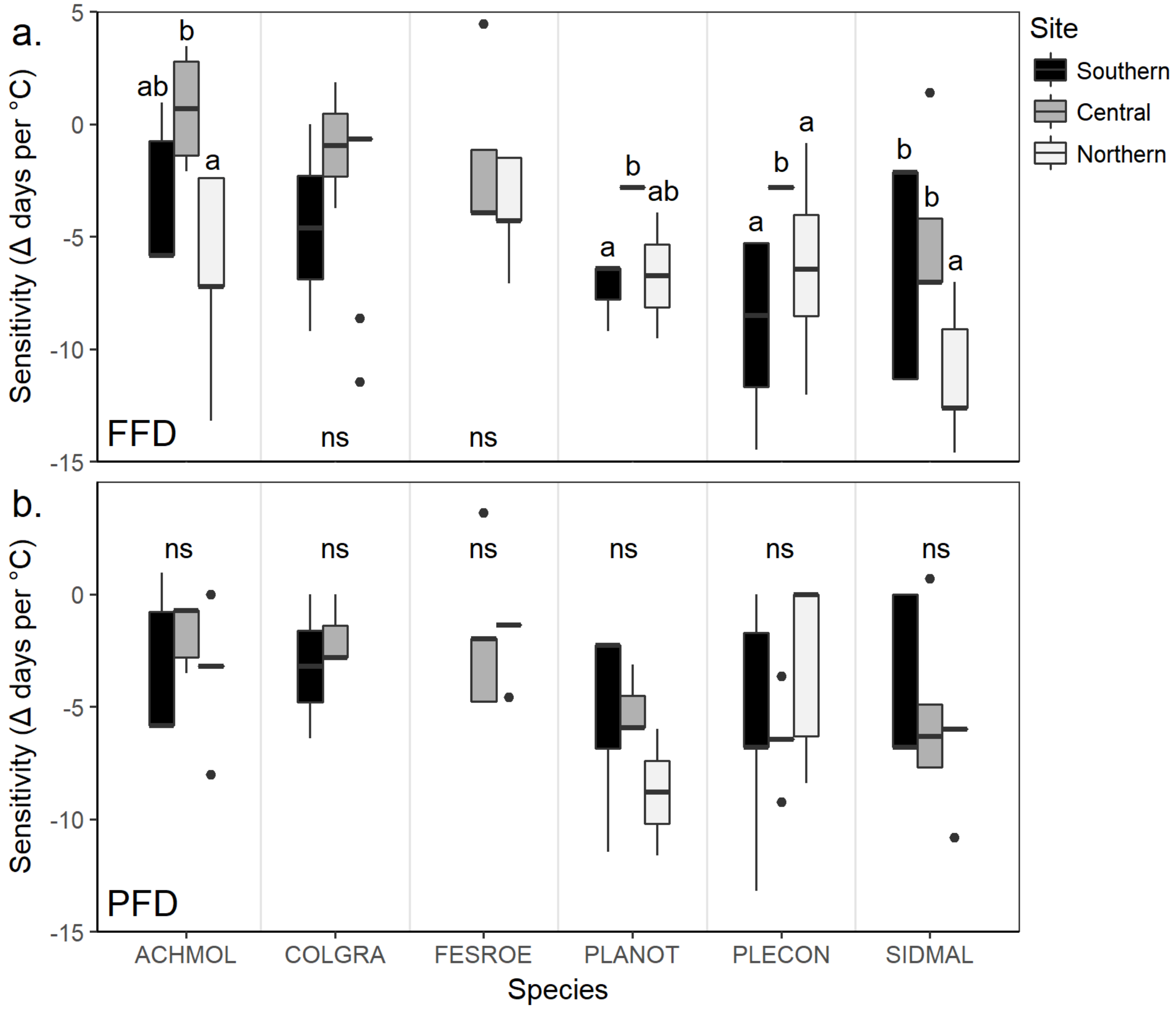

Figure S3. Temperature sensitivities between warmed and ambient plots for (a) first flower date (FFD) and (b) peak flower date (PFD) across sites. Different letters indicate significant or marginal differences between sites within a species ($p < 0.10$; Tukey's post-hoc comparisons); 'ns' = not significant.

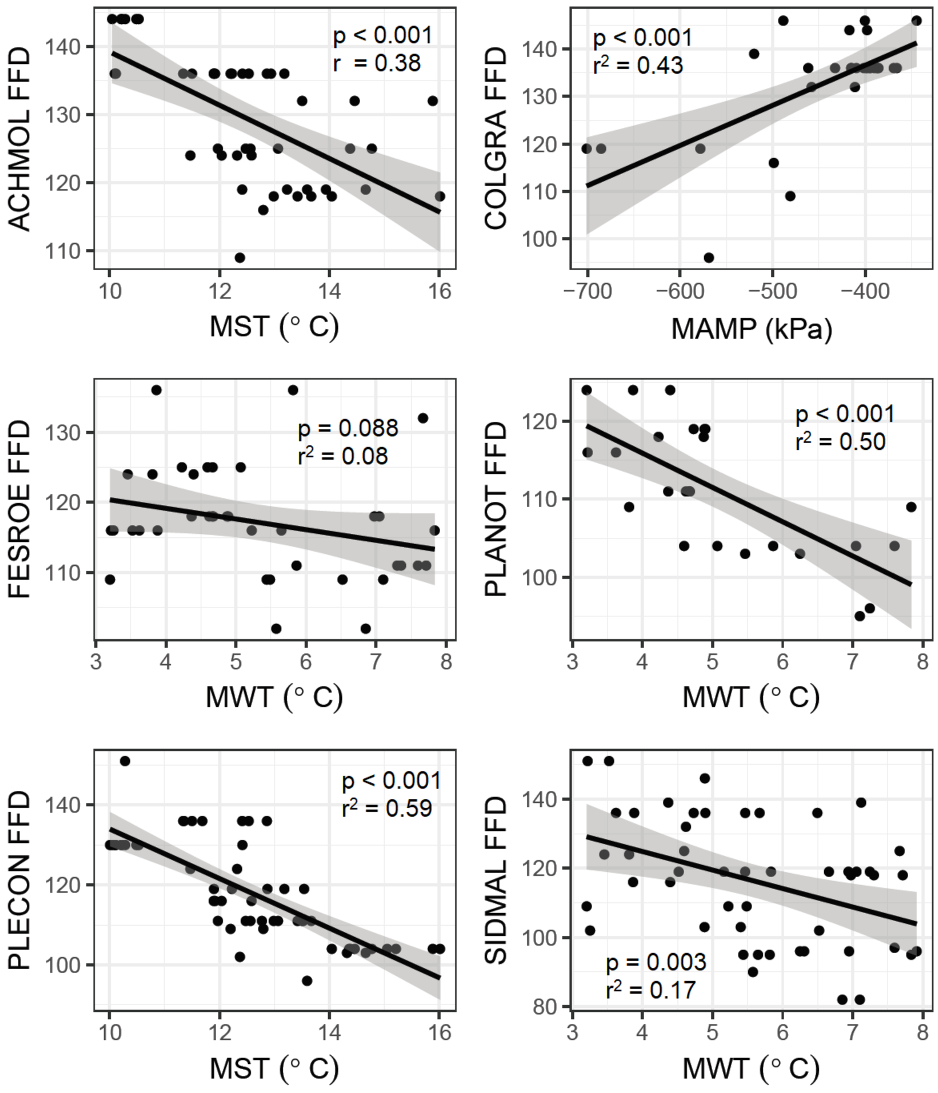

Figure S4. First flower dates (FFD) regressed against each species' most important predictor variable (Table 2). MST = 'mean spring temperature', MAMP = 'mean annual matric potential',

MWT = ‘mean winter temperature’.

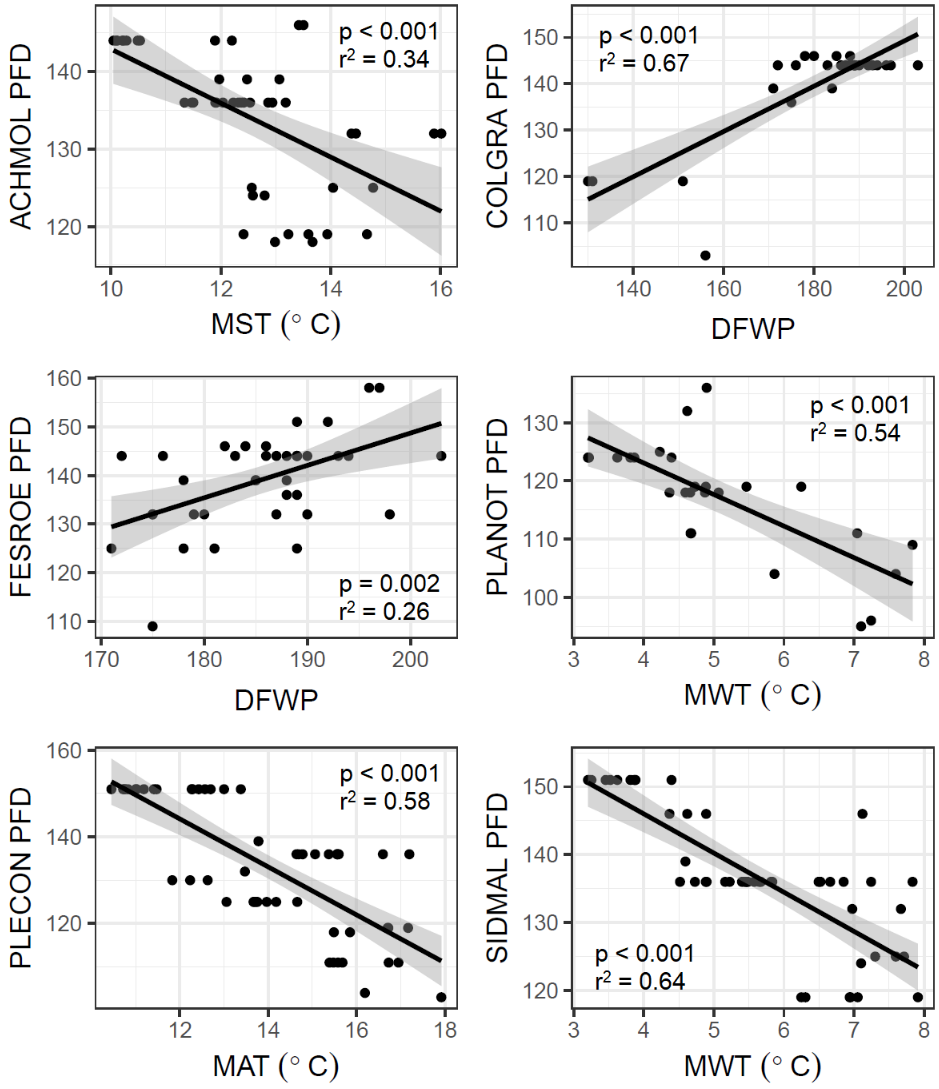

Figure S5. Peak flower dates (PFD) regressed against each species’ most important predictor variable (Table 2). MST = ‘mean spring temperature’, DFWP = ‘date of first wilting point’,

MWT = ‘mean winter temperature’, MAT = ‘mean annual temperature’.

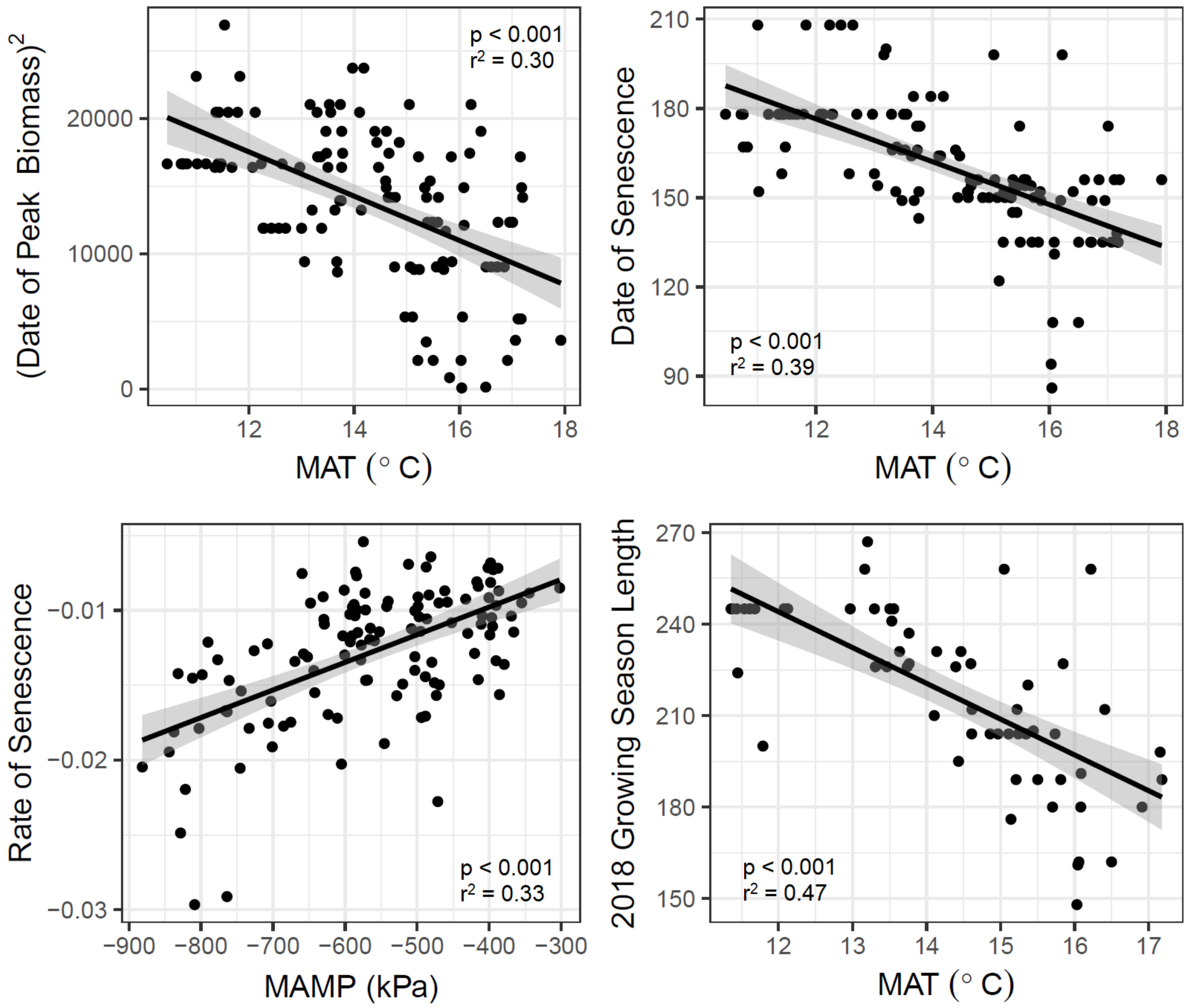

Figure S6. Community-level phenology variables each regressed against their most important predictor variable (Table 2). Date values are in Julian days; the date of peak biomass was squared to improve normality. MAT = ‘mean annual temperature’, MAMP = ‘mean annual matric potential (adjusted)’.

	Experimental Site		
	Southern	Central	Northern
Land Management	Siskiyou Field Institute	The Nature Conservancy	Capitol Land Trust
Latitude; Longitude	42.27811; -123.642278	44.02615; -123.182171	46.86415; -122.958918
Elevation (m)	394	165	79
Monthly Air Temp (°C) (PRISM)			
Mean	12.3	11.4	10.6
Max	20.2	17.3	15.9
Min	4.4	5.4	5.3
Annual Precipitation (mm) (PRISM)	1434	1134	1240
Fall (Sep-Nov)	316	293	363
Winter (Dec-Feb)	742	496	499
Spring (Mar-May)	331	275	288
Summer (Jun-Aug)	45	71	90
Soil			
Taxonomy	Loamy-skeletal, mixed, superactive, mesic Entic Ultic Haploxerolls	Very-fine, smectitic, mesic Vertic Haploxerolls	Medial, mixed, mesic Typic Haploxerands
Series	Takilma cobbly loam	Hazelair silty clay loam	Cathcart medial-loam pasture

Table S1. Experimental site information. PRISM model is from the period 1981-2010 (http://www.prism.oregonstate.edu/). Soil taxonomy and series information: Natural Resources Conservation Service, United States Department of Agriculture (USDA), Web Soil Survey (http://websoilsurvey.nrcs.usda.gov/).

**Abundances**		**ACHMOL**		**COLGRA**		**FESROE**		**MICLAC**		**PLANOT**		**PLECON**		**RANAUS**		**SIDMAL**	
	df	LR X^2	*p*	LR X^2	*p*	LR X^2	*p*	LR X^2	*p*	LR X^2	*p*	LR X^2	*p*	LR X^2	*p*	LR X^2	*p*
Model Type		Negative Binomial		Negative Binomial		Poisson		Negative Binomial		Negative Binomial		Zero-Inflated Negative Binomial		Negative Binomial		Negative Binomial	
**2-way Analysis of Deviance**																	
Site	2	30.9	**<0.001**	1357.6	**<0.001**	1.5	0.227	-	-	15.5	**<0.001**	67.8	**<0.001**	-	-	333.6	**<0.001**
Climate Trt	3	9.5	**0.024**	5.2	0.156	4.1	0.252	-	-	14.2	**0.003**	2.3	0.513	-	-	0.8	0.854
Site x Climate Trt	6	13.2	**0.040**	22.9	**0.001**	2.0	0.574	-	-	4.5	0.605	20.9	**0.002**	-	-	29.3	**<0.001**
**1-way Analysis of Deviance: within sites**																	
Southern: Climate Trt	3	2.1	0.544	1.4	0.717	-	-	-	-	2.7	0.433	1.1	0.774	-	-	14.1	**0.003**
Central: Climate Trt	3	7.8	*0.051*	33.2	**0.000**	0.3	0.965	-	-	11.4	**0.010**	31.1	**<0.001**	-	-	2.6	0.462
Northern: Climate Trt	3	11.7	**0.008**	7.0	*0.073*	6.6	*0.086*	4.9	0.177	5.7	0.126	9.9	**0.019**	12.3	**0.007**	3.4	0.336

Table S2. Likelihood Ratio (LR) χ^2 values, degrees of freedom (df), and p-values from analyses of abundances of reproductive plants of our eight focal species. 'Climate trt' indicates climate treatment, and '-' indicates not enough data to run a statistical test. Bold = significant at p < 0.05, italics = marginally significant (p < 0.10).

FFD	df	ACHMOL *p*	COLGRA *p*	FESROE *p*	MICLAC *p*	PLANOT *p*	PLECON *p*	RANAUS *p*	SIDMAL *p*
**2-way ANOVAs (using climate treatments)**		Error df = 33	Error df = 18	Error df = 30	-	Error df = 14	Error df = 42	-	Error df = 37
Site	2	**0.001**	**<0.001**	0.351	-	0.491	**<0.001**	-	**0.020**
Climate Trt	3	**0.019**	0.172	**0.020**	-	**0.001**	**<0.001**	-	**<0.001**
Site x Climate Trt	6	**0.028**	0.214	**0.038**	-	0.244	**0.028**	-	0.262
**2-way ANOVAs (using warming treatments)**		Error df = 39	Error df = 24	Error df = 34	-	Error df = 20	Error df = 48	-	Error df = 43
Site	2	**0.001**	**<0.001**	0.405	-	0.507	**<0.001**	-	**0.010**
Warming	1	**0.001**	*0.075*	**0.013**	-	**<0.001**	**<0.001**	-	**<0.001**
Site x Warming	2	**0.006**	0.672	0.700	-	*0.084*	**0.005**	-	0.145
**1-way ANOVAs (site effects, ambient plots)**		Error df = 20	Error df = 12	Error df = 18	-	Error df = 15	Error df = 26	-	Error df = 21
Site	2	**<0.001**	**<0.001**	0.681	-	**0.017**	**<0.001**	-	0.437
**1-way ANOVAs Southern**		Error df = 9	-	-	-	Error df = 2	Error df = 11	-	Error df = 15
Climate Trt	3	*0.079*	-	-	-	*0.068*	**0.007**	-	0.143
**1-way ANOVAs Central**		Error df = 10	Error df = 2	Error df = 14	-	Error df = 8	#	-	Error df = 6
Climate Trt	3	0.952	0.928	0.170	-	0.189	#	-	*0.073*
**1-way ANOVAs Northern**		Error df = 14	Error df = 16	Error df = 16	Error df = 8	Error df = 4	Error df = 16	Error df = 13	Error df = 16
Climate Trt	3	**0.007**	*0.071*	**0.014**	*0.052*	0.118	**0.001**	**0.002**	**0.001**
**t-tests: Southern**		df = 9.1	df = 1	-	-	df = 2	df = 13	-	df = 17
Warming		*0.070*	0.500	-	-	**0.016**	**<0.001**	-	**0.018**
**t-tests: Central**		df = 9.3	df = 3.9	df = 16	-	df = 10	#	-	df = 8
Warming		0.585	0.725	*0.058*	-	**0.039**	#	-	*0.070*
**t-tests: Northern**		df = 12.2	df = 11	df = 18	df = 7	df = 6	df = 18	df = 15	df = 18
Warming		**<0.001**	*0.089*	*0.077*	**0.003**	**0.025**	**<0.001**	**0.002**	**<0.001**

Table S3. Degrees of freedom (df) and p-values from ANOVAs and t-tests of first flower date (FFD) of our eight focal species. '-' indicates not enough data to run a statistical test. '#' indicates that tests for PLECON could not be run at the central site because there was no variance among all ambient plots nor among all warmed plots, Bold = significant at $p < 0.05$, italics = marginally significant ($p < 0.10$).

PFD		ACHMOL	COLGRA	FESROE	MICLAC	PLANOT	PLECON	RANAUS	SIDMAL
	df	*p*	*p*	*p*	*p*	*p*	*p*	*p*	*p*
**2-way ANOVAs (using climate treatments)**		Error df = 33	Error df = 18	Error df = 30	-	Error df = 14	Error df = 42	-	Error df = 37
Site	2	**<0.001**	**<0.001**	**0.002**	-	0.321	**<0.001**	-	**<0.001**
Climate Trt	3	**0.037**	**<0.001**	0.125	-	**0.003**	**<0.001**	-	**<0.001**
Site x Climate Trt	6	0.656	**<0.001**	0.825	-	0.183	0.173	-	0.543
**2-way ANOVAs (using warming treatments)**		Error df = 39	-	Error df = 34	-	Error df = 20	Error df = 48	-	Error df = 43
Site	2	**0.001**	-	**0.001**	-	0.328	**<0.001**	-	**<0.001**
Warming	1	**0.002**	-	**0.016**	-	**<0.001**	**<0.001**	-	**<0.001**
Site x Warming	2	0.606	-	0.720	-	0.422	*0.080*	-	0.325
**1-way ANOVAs (site effects, ambient plots)**		Error df = 20	-	Error df = 18	-	Error df = 15	Error df = 26	-	Error df = 21
Site	2	**0.022**	-	**0.003**	-	0.184	**<0.001**	-	**<0.001**
**1-way ANOVAs Southern**		Error df = 9	-	-	-	Error df = 2	Error df = 11	-	Error df = 15
Climate Trt	3	*0.079*	-	-	-	0.650	**0.032**	-	**0.005**
**1-way ANOVAs Central**		Error df = 10	Error df = 2	Error df = 14	-	Error df = 8	Error df = 15	-	Error df = 6
Climate Trt	3	0.868	0.506	0.410	-	**0.015**	**<0.001**	-	0.149
**1-way ANOVAs Northern**		Error df = 14	-	Error df = 16	Error df = 8	-	Error df = 16	Error df = 13	Error df = 16
Climate Trt	3	**0.032**	-	0.288	0.389	-	0.261	**<0.001**	**<0.001**
**t-tests: Southern**		df = 9.1	df = 1	-	-	df = 3.7	df = 5	-	df = 8
Warming		*0.070*	0.500	-	-	0.240	**0.041**	-	**0.004**
**t-tests: Central**		df = 8.7	df = 2	df = 16	-	df = 5.8	df = 17	-	df = 8
Warming		0.377	0.184	*0.091*	-	**0.008**	**<0.001**	-	**0.016**
**t-tests: Northern**		df = 9	-	df = 17	df = 7	df = 6	df = 9	df = 14.7	df = 8
Warming		**0.003**	-	*0.079*	**0.033**	**0.001**	*0.081*	**<0.001**	**<0.001**

Table S4. Degrees of freedom (df) and p-values from ANOVAs and t-tests of peak flower date (PFD) for each of our eight focal species. '-' indicates not enough data to run a statistical test. '@' indicates that PFD data for *C. grandiflora* at the northern site were excluded due to an overwhelmingly large sample size (> 500 plants per plot) which made it logistically impossible to count flowers during its peak growing period. Bold = significant at p < 0.05, italics = marginally significant (p < 0.10).

**Sensitivity (FFD)**		**ACHMOL**	**COLGRA**	**FESROE**	**PLANOT**	**PLECON**	**SIDMAL**
	df	*p*	*p*	*p*	*p*	*p*	*p*
**1-way ANOVAs (site effect on sensitivity)**		Error df = 19	Error df = 12	Error df = 16	Error df = 5	Error df = 22	Error df = 22
Site	2	**0.004**	0.632	0.660	*0.086*	**0.002**	**0.013**
**Sensitivity (PFD)**		**ACHMOL**	**COLGRA**	**FESROE**	**PLANOT**	**PLECON**	**SIDMAL**
	df	*p*	*p*	*p*	*p*	*p*	*p*
**1-way ANOVAs (site effect on sensitivity)**		Error df = 19	Error df = 3	Error df = 16	Error df = 5	Error df = 22	Error df = 22
Site	2	0.353	0.652	0.675	0.561	*0.096*	0.322

Table S5. Degrees of freedom (df) and p-values from ANOVAs of temperature sensitivities (difference between warmed and ambient plots divided by 2.5°C) for first flowering date (FFD) and peak flowering date (PFD) across sites for six focal species.

Phenology Variable	Model(s)	K	AIC_c	δAIC_c	ω	Adj. R^2
FFD (2017)						
ACHMOL	–MST	1	313.8	0.00	0.51	0.37
COLGRA	MAMP	1	220.2	0.00	0.35	0.41
FESROE	–MWT – DFWP	2	265.1	0.00	0.40	0.15
PLANOT	–MWT	1	171.9	0.00	0.54	0.47
PLECON	–MST – DFWP	2	374.2	0.00	0.44	0.66
	–MST – MAMP	2	375.9	1.76	0.27	0.65
SIDMAL	–MWT	1	418.0	0.00	0.38	0.16
PFD (2017)						
ACHMOL	–MST + DFWP	2	308.7	0.00	0.28	0.39
	–MAT	2	308.9	0.16	0.24	0.37
	–MST + MAMP	2	309.8	1.03	0.15	0.37
COLGRA	DFWP	1	198.2	1.79	0.14	0.66
FESROE	–MAT + DFWP	2	269.2	0.00	0.36	0.38
	–MST + DFWP	2	269.8	0.68	0.40	0.36
PLANOT	–MWT – DFWP	2	177.5	0.00	0.42	0.57
	–MWT	1	178.5	1.01	0.26	0.52
PLECON	–MAT + DFWP	2	384.0	0.00	0.82	0.71
SIDMAL	–MWT + MAMP	2	311.5	0.00	0.54	0.71
	–MWT + DFWP	2	312.1	0.55	0.41	0.70
NDVI (2017+2018):						
Date of peak biomass	–MAT + DFWP	2	2374.1	0.00	0.57	0.32
	–MAT + MAMP	2	2375.8	1.68	0.25	0.31
Date of senescence	–MAT + MAMP	2	1016.1	0.00	0.59	0.44
	–MAT – DBWP	2	1017.5	1.39	0.30	0.43
Rate of senescence	–MAT + MAMP	2	-1006.5	0.00	0.60	0.35
GSL (2018)	–MAT + MAMP	2	523.9	0.00	0.44	0.57
	–MAT – DBWP	2	524.2	0.29	0.38	0.57

Table S6. Candidate models to describe the phenology response variables (FFD = first flowering date, PFD = peak flowering date, GSL = growing season length). K = the number of parameters, AIC_c = small-sample-size corrected version of Akaike Information Criterion, δAIC_c = change in AIC_c, ω = Akaike's weight. Only models with $\delta AIC_c < 2$ are reported, and two-parameter models are not reported if they had an AIC_c less than a model including one of its parameters alone. Temperature variables: MAT = mean annual temp, MWT = mean winter temp, MST = mean spring temp; moisture variables: MAMP = mean annual matric potential (adjusted so values < -1,500 became -1,500), DFWP = date of first wilting point, DBWP = days below wilting point.

		Date of peak biomass	Date of senescence	Rate of senescence	Growing Season Length
	df	*p*	*p*	*p*	*p*
**3-Way Repeated Measures ANOVAs (2017 + 2018)**		Error df = 108	Error df = 108	Error df = 108	NA
Site	2	**<0.001**	**<0.001**	**<0.001**	
Warming	1	**<0.001**	**0.002**	0.666	
Site x Warming	2	0.349	**0.023**	0.339	
Year	1	0.701	**0.002**	**0.009**	
Site x Year	2	*0.096*	0.187	**0.001**	
Warming x Year	1	0.288	**0.011**	0.574	
Site x Warming x Year	2	0.565	0.659	0.168	
**1-Way Repeated Measures ANOVAs* (year effects within sites)**		Error df = 19	Error df = 9*	Error df = 9*	NA
**Southern: Year**	1	0.164	**0.008**	**0.010**	
**Central: Year**	1	0.440	0.975	0.762	
**Northern: Year**	1	**0.019**	0.337	0.163	
**2-way ANOVAs: 2018**		Error df = 54	Error df = 54	Error df = 54	Error df = 54
Site	2	**<0.001**	**<0.001**	0.172	**<0.001**
Warming	1	**0.002**	**<0.001**	0.504	**0.008**
Site x Warming	2	0.408	0.288	0.908	*0.076*
**1-way ANOVAs: 2018 (site effects, ambient plots)**		NA	NA	NA	Error df = 27
Site	2				**<0.001**
**2-way ANOVAs: 2017**		Error df = 54	Error df = 54	Error df = 54	NA
Site	2	**<0.001**	**<0.001**	**<0.001**	
Warming	1	**<0.001**	0.317	0.894	
Site x Warming	2	0.623	**0.013**	**0.045**	
**1-way ANOVAs: 2017 (site effects, ambient plots)**		NA	Error df = 27	Error df = 27	NA
Site	2		**0.013**	**<0.001**	
**t-tests: Southern 2018**		NA	NA	NA	df = 14.4
Warming					**0.004**
**t-tests: Central 2018**		NA	NA	NA	df = 14.5
Warming					**0.037**
**t-tests: Northern 2018**		NA	NA	NA	df = 18.0
Warming					0.965
**t-tests: Southern 2017**		NA	df = 9.0	df = 17.9	NA
Warming			**0.015**	0.163	
**t-tests: Central 2017**		NA	df = 16.0	df = 17.9	NA
Warming			**0.049**	**0.029**	
**t-tests: Northern 2017**		NA	df = 16.0	df = 17.6	NA
Warming			0.172	0.881	

Table S7. Results from statistical analyses of community-level phenology response variables. *Year effects within sites used ambient-only plots for date and rate of senescence.
